# Selective Inhibitors of a Human Prolyl Hydroxylase (OGFOD1) Involved in Ribosomal Decoding

**DOI:** 10.1002/chem.201804790

**Published:** 2019-01-08

**Authors:** Cyrille C. Thinnes, Christopher T. Lohans, Martine I. Abboud, Tzu‐Lan Yeh, Anthony Tumber, Radosław P. Nowak, Martin Attwood, Matthew E. Cockman, Udo Oppermann, Christoph Loenarz, Christopher J. Schofield

**Affiliations:** ^1^ Department of Chemistry University of Oxford Oxford OX1 3TA UK; ^2^ Structural Genomics Consortium University of Oxford Headington OX3 7DQ UK; ^3^ Department of Cancer Biology Dana-Farber Cancer Institute Boston, MA 02215 USA; ^4^ Centre for Cellular and Molecular Physiology University of Oxford Oxford OX3 7BN UK; ^5^ Institute of Pharmaceutical Sciences Albert-Ludwigs-Universität Freiburg 79104 Freiburg Germany

**Keywords:** barbiturate, epigenetics, histone demethylases, inhibitors, medicinal chemistry, OGFOD1, 2-oxoglutarate oxygenase

## Abstract

Human prolyl hydroxylases are involved in the modification of transcription factors, procollagen, and ribosomal proteins, and are current medicinal chemistry targets. To date, there are few reports on inhibitors selective for the different types of prolyl hydroxylases. We report a structurally informed template‐based strategy for the development of inhibitors selective for the human ribosomal prolyl hydroxylase OGFOD1. These inhibitors did not target the other human oxygenases tested, including the structurally similar hypoxia‐inducible transcription factor prolyl hydroxylase, PHD2.

## Introduction

**Figure 1:**
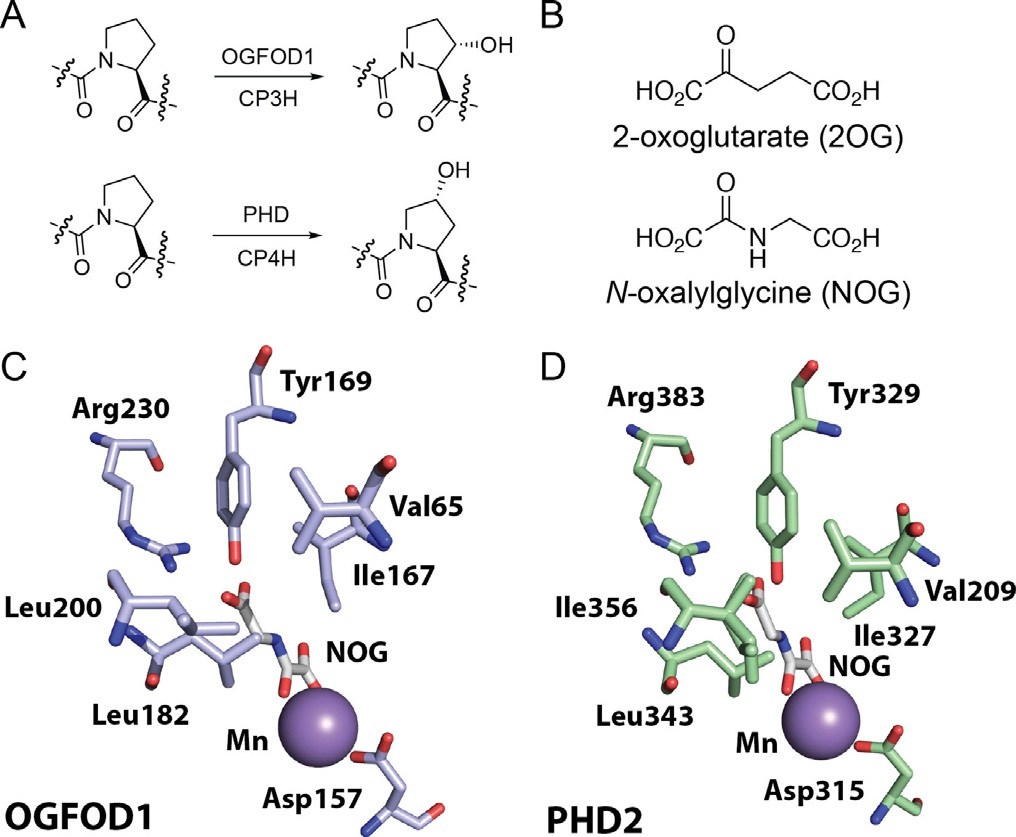
Prolyl hydroxylase reactions and structures: (A) Regio‐ and stereoselectivity of hydroxylations catalyzed by different types of prolyl hydroxylases. Each hydroxylation is coupled to the conversion of 2‐oxoglutarate (2OG) and O_2_ into succinate and CO_2_. OGFOD1 acts on a ribosomal protein, the CPHs act on procollagen, and the PHDs act on the hypoxia inducible factor (HIF) transcription factors.^5^ (B) Structures of 2OG and the 2OG analogue *N*‐oxalylglycine (NOG). (C, D) Views from the crystallographically observed active sites of OGFOD1 (PDB 4NHX)^10^ and PHD2 (PDB 5L9R)^15^ showing the interactions between active site residues, the bound metal [Mn^II^ substituting for Fe^II^], and NOG.

In humans and other animals, prolyl hydroxylases (PHs) play critical roles in collagen biosynthesis and hypoxia sensing.^1^, ^2^, ^3^ The PHs are Fe^II^ and 2‐oxoglutarate (2OG) dependent oxygenases, which normally produce succinate and CO_2_ as coproducts.^4^ The procollagen PHs (CP3H and CP4 H) hydroxylate either C3 or C4 of prolyl residues; the latter is essential for maintenance of the collagen triple helix secondary structure (Figure 1 A).^5^, ^6^ In humans, the prolyl hydroxylase domain enzymes (PHD1, 2, and 3) act as oxygen sensors in the chronic response to hypoxia by catalyzing oxygen‐limited hydroxylation of prolyl residues in the hypoxia‐inducible factor‐α (HIFα) subunits of HIF transcription factors, leading to HIFα degradation in aerobic conditions (Figure 1 A).^1^

The human 2OG oxygenase OGFOD1 has been recently shown to hydroxylate Pro‐62 of the ribosomal protein RPS23.^7^, ^8^, ^9^, ^10^ Pro‐62_RPS23_ is situated in the ribosomal decoding site, which is responsible for ensuring the fidelity of mRNA codon recognition by tRNA and release factor proteins during protein synthesis.^11^, ^12^ While the role of this hydroxylation in human and animal cells is not yet understood, the *Saccharomyces cerevisiae* OGFOD1 homologue Tpa1p is proposed to catalyze dihydroxylation of the corresponding prolyl residue, and to regulate translational accuracy in an mRNA sequence context‐dependent manner.^8^, ^13^, ^14^

Of the ∼60–70 human 2OG oxygenases, some are current medicinal chemistry targets, including enzymes involved in chromatin modification and lipid metabolism.^5^, ^16^ Inhibition of the procollagen hydroxylases is under consideration as a target to limit the overproduction of collagen associated with certain cancers and fibrotic diseases.^17^ The PHDs are presently being targeted for the treatment of hypoxia‐related diseases, with inhibitors in late‐stage clinical trials for anaemia.^18^ If OGFOD1 is indeed involved in mRNA codon recognition, as suggested based on studies of yeast homologues,^8^, ^13^ small‐molecule‐mediated inhibition of ribosomal hydroxylation could prove useful for the treatment of diseases such as muscular dystrophy that are caused by premature stop‐codons through nonsense suppression.^19^ However, due to the uncertainty regarding the specific roles of OGFOD1 and OGFOD1‐catalysed hydroxylation in animals, it is unclear how exactly its inhibition might manifest. Thus, such OGFOD1 inhibitors are also of interest as chemical probes to decipher the biological role of RPS23 hydroxylation, as well as those of other recently reported ribosome‐associated hydroxylations.^20^, ^21^, ^22^, ^23^

As the 2OG oxygenases are involved in diverse biological processes, developing inhibitors selective for particular oxygenases is an important therapeutic consideration. The available biophysical evidence, principally from crystallography, implies that key features in the active sites of the different types of human PHs are substantially, but not completely, conserved.^4^ Therefore, there is the potential that inhibitors targeting OGFOD1 could also interfere with hypoxia sensing and collagen biosynthesis through inhibition of other PHs (or vice versa). To date, no detailed evidence for inhibitors selective for the different types of human PHs have been reported. Here, we establish the viability of a structure‐guided template‐based approach for the development of selective OGFOD1 inhibitors which do not target the other human oxygenases tested, including the human hypoxia‐sensing enzyme PHD2.

## Results

To assess the viability of developing inhibitors selective for particular PHs, with a focus on OGFOD1, we first compared crystal structures of OGFOD1 and PHD2.^10^, ^24^, ^25^ Although there are differences in the OGFOD1 and PHD2 active sites, the binding modes of 2OG [and the 2OG analogue *N*‐oxalylglycine (NOG)] appear largely conserved (Figure 1 B–D). The 2‐oxoacid group of 2OG (or NOG) binds to the metal in a bidentate manner, while the 2OG C‐5 carboxylate is positioned to interact with conserved tyrosine and arginine residues (Tyr169 and Arg230 in OGFOD1, Tyr329 and Arg383 in PHD2; Figure 1 C, D). These comparisons, along with those shown for other human oxygenases,^4^ suggest that inadvertent inhibition of the PHDs may represent a challenge in developing selective OGFOD1 inhibitors, and vice versa.

**Figure 2:**
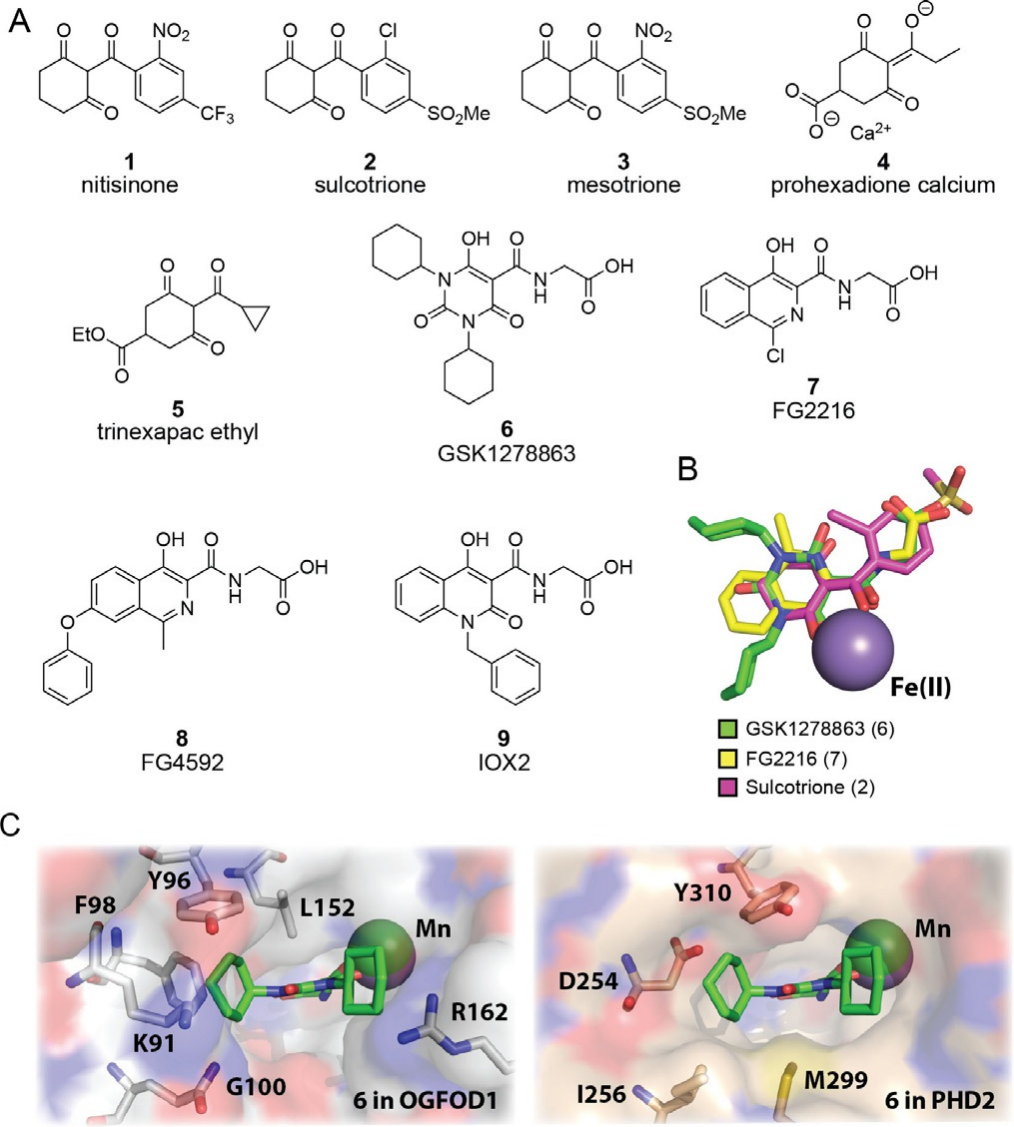
Oxygenase inhibitors and their binding modes: (A) Structures of inhibitors of 4‐hydroxyphenylpyruvate dioxygenase (HPPD) and PHD2. Like OGFOD1 and PHD2, HPPD is an Fe^II^‐dependent 2‐oxoacid oxygenase, but from a different structural family. (B) Comparison of the common chelation motifs of sulcotrione **2**, GSK1278863 **6**, and FG2216 **7**. The structures of sulcotrione **2** and GSK1278863 **6** were modeled onto the crystallographically observed structure of PHD2 with bound FG2216 **7** (PDB 3HQU).^25^ A larger version is shown in Figure S5 in the Supporting Information. (C) In silico binding model of GSK1278863 **6** with OGFOD1 (PDB 4NHX)^10^ and PHD2 (PDB 5L9R).^15^ The tricarbonyl is expected to interact with the bound metal, while the *N*‐cyclohexyl groups likely occupy the substrate binding position.

Given the conserved elements of the OGFOD1 and PHD2 active sites,^10^ and evidence for induced fit and conformational movements in prolyl hydroxylase catalysis,^15^ we contemplated a structurally informed template‐derivatization approach for developing OGFOD1 inhibitors.^26^ We first considered the 4‐hydroxyphenylpyruvate dioxygenase (HPPD) inhibitor nitisinone **1** (which is clinically used for the treatment of tyrosinaemia)^27^ and related plant growth inhibitors sulcotrione **2**, mesotrione **3**, prohexadione‐calcium **4**, and trinexapac‐ethyl **5** (Figure 2 A).^28^ These compounds are related to the “tricarbonyl” chemotype found in the PHD inhibitor GSK1278 863 **6**, which is currently in clinical trials for the treatment of anaemia.^18^ In addition to GSK1278863 **6**, we tested PHD inhibitors FG2216 **7**, FG4592 **8**, and IOX2 **9**.^29^, ^30^

These inhibitors were screened for binding to OGFOD1 and PHD2 by differential scanning fluorimetry (DSF) and for inhibition using matrix‐assisted laser desorption ionization time‐of‐flight mass spectrometry (MALDI‐TOF MS) (Figures S1–S4, Supporting Information).^31^, ^32^ As expected based on previous studies,^33^ PHD inhibitors FG2216 **7**, FG4592 **8**, and IOX2 **9** inhibited OGFOD1 (Figure S1). Notably, triketone‐based HPPD and plant growth inhibitors **1**–**5** (Figure 2 A), related to GSK1278863 **6**, displayed moderate inhibition of OGFOD1, while not inhibiting the activity of PHD2 within our limits of detection (Figure S2). Additionally, these inhibitors increased the apparent thermal stability of OGFOD1, as observed by DSF (Figures S3, S4). The inhibition of OGFOD1 by prohexadione‐calcium **4** and trinexapac‐ethyl **5** (Figure S2) suggests that further investigation of these agrochemicals is warranted, given their potential interactions with other human 2OG oxygenases.

GSK1278863 **6** and sulcotrione **2** were modeled into the crystallographically observed OGFOD1 and PHD2 active sites, with consideration for potential metal‐chelating properties, salt bridge interactions, and crystallographic studies of PHD2 with inhibitors (such as FG2216 **7**; PDB 3HQU) (Figure 2 B and Figure S5).^25^ These analyses imply that both **6** and **2** will engage in Fe^II^ chelation via an enolate form of their 1,3‐diketone motif (Figure S5). Similarly to the C‐5 carboxylate of 2OG/NOG,^10^ and the carboxylate of FG2216 **7**,^25^ the GSK1278863 **6** carboxylate is predicted to interact with Tyr169 and Arg230 of OGFOD1. The OGFOD1:**6** model suggests that the inhibitor ring systems bind in two approximately perpendicular planes, one comprising the diketone ring and glycinamide side chain, and the other formed from the cyclohexyl rings (Figure 2 C). The model also implies that the cyclohexyl rings engage differently with the OGFOD1 and PHD2 active sites (Figure 2 C). By contrast, compounds based on an isoquinoline chemotype (e.g., FG2216 **7**) are predicted to bind in a more co‐planar manner^25^ and were considered less likely to readily lead to OGFOD1‐selective inhibitors.

While many reported PHD2 inhibitors possess a glycinamide side chain, the triketone plant growth/HPPD inhibitors do not (Figure 2 A). Modeling results suggest that the sulcotrione **2** methyl sulfonyl group and the nitro group of mesotrione **3** and nitisinone **1** may mimic the 2OG/glycinamide side‐chain binding at their active sites (Figure 2 B). It is possible that the enzyme active site may accommodate the side chains of nitisinone **1**, sulcotrione **2**, and mesotrione **3**, which are bulkier than the glycinamide side chain of FG2216 **7**; however, in the absence of structural information, we cannot preclude the possibility that these inhibitors with bulky side chains interact with the enzyme in an alternate orientation.

Both the barbiturate‐based PHD inhibitors (e.g., **6**) and the HPPD/plant growth inhibitors (e.g., **1**–**5**) have a tri‐carbonyl motif. However, triketones **1**–**5** are likely more conformationally flexible than the barbiturates. Notably, the DSF results suggest that the triketones stabilize OGFOD1 more than PHD2 (Figure S3), whereas the glycinamide‐containing PHD2 inhibitors (e.g., **6**–**9**) stabilize PHD2 more than OGFOD1 (Figure S4). We thus explored whether modification of the barbiturate/cyclohexane‐1,3‐dione‐based ring scaffolds could be exploited in the development of selective OGFOD1 inhibitors.

**Figure 3:**
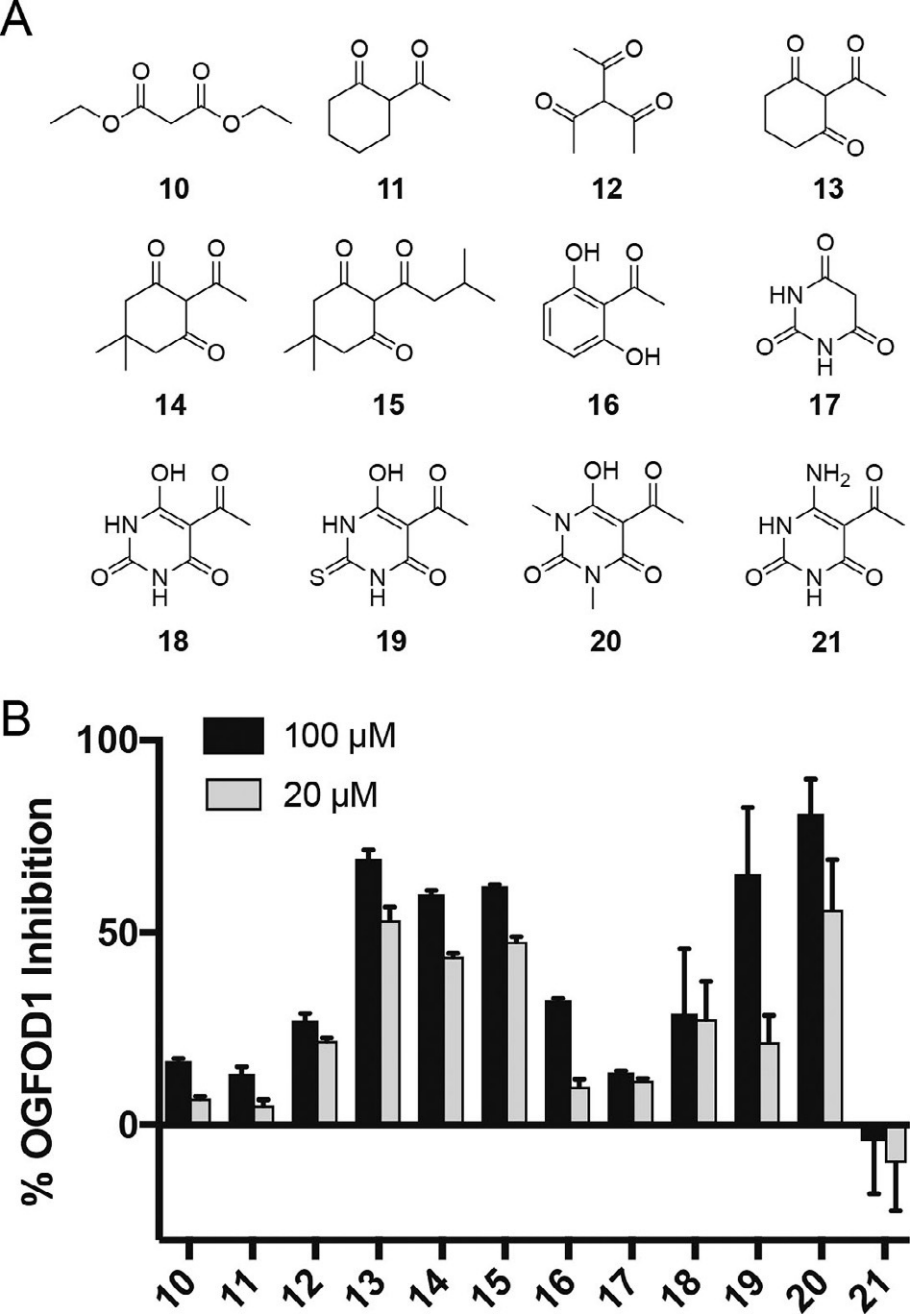
Fragment‐based screening approach for OGFOD1 inhibition: (A) Structures of diketones, triketones, and structurally related compounds used for fragment‐based screening. (B) Inhibitory effect of the fragments on the hydroxylation activity of OGFOD1 (1 μm). The plotted data represent the mean percentage inhibition for the experiment performed in triplicate, whereas the error bars indicate the standard deviation.

**Figure 4:**
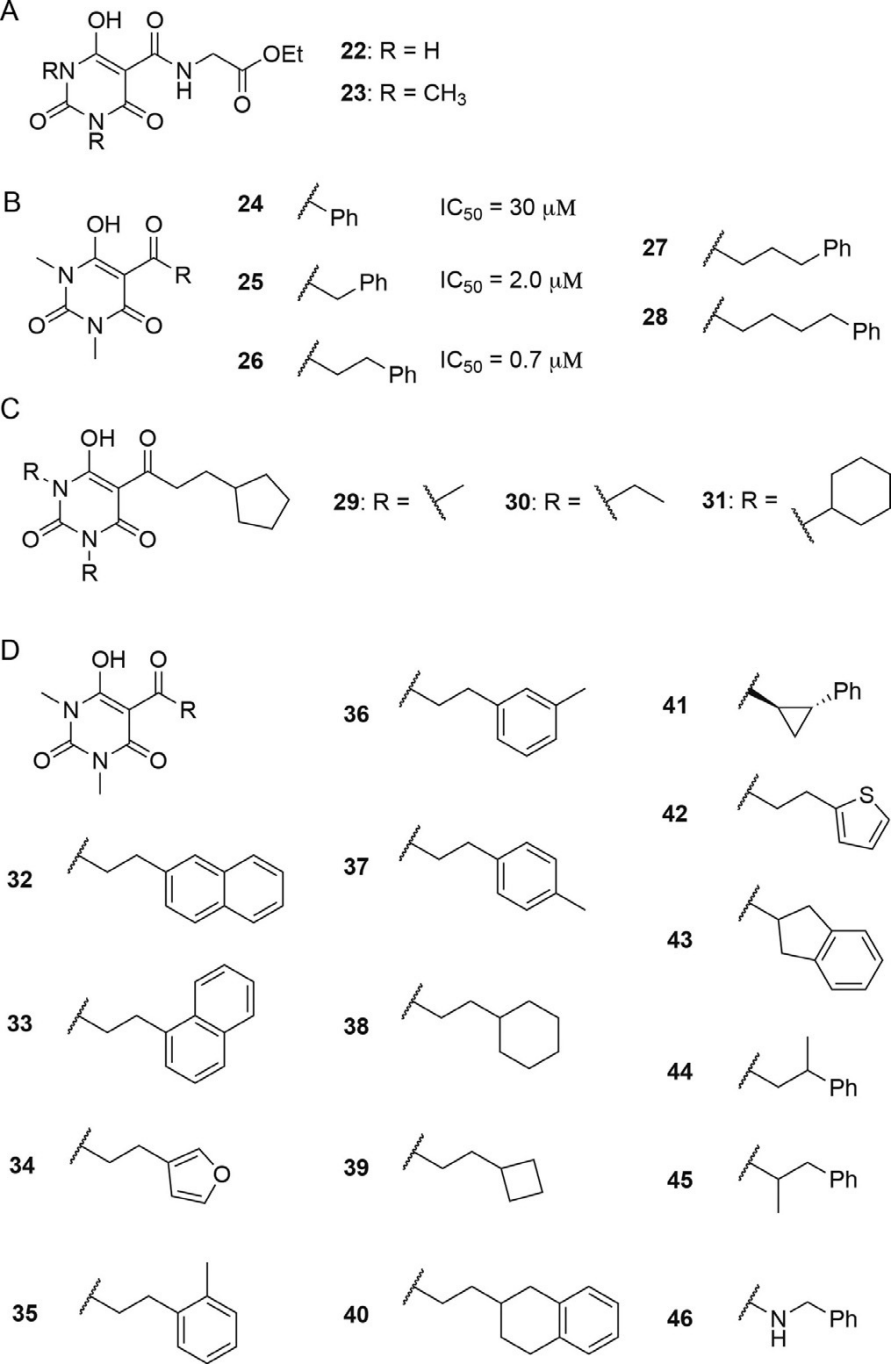
Optimization studies of barbiturate inhibitors: (A) Structures of the barbiturate glycinamide ethyl esters tested against OGFOD1 and PHD2. (B) Panel of phenyl‐substituted *N*,*N′*‐dimethylbarbiturates, demonstrating the impact of increasing acyl chain length on OGFOD1 inhibition. (C) Panel of barbiturates synthesized with different *N*‐alkyl substituents. (D) Panel of C‐5 substituted *N*,*N′*‐dimethylbarbiturates based on lead compound **CCT3** (**26**).

Di‐carbonyl compounds (e.g., diethyl malonate **10** and 2‐acetylcyclohexanone **11**), triketones (e.g., triacetylmethane **12** and substituted 1,3‐cyclohexanediones **13**–**15**), and structurally related compounds (e.g., 2′,6′‐dihydroxyacetophenone **16** and barbituric acid **17**) were screened for OGFOD1 inhibition (Figure 3). The preliminary results suggested that the degree of OGFOD1 inhibition may in part relate to the propensity for inhibitor enolization. Thus, whereas the diketones tested (i.e., **10**, **11**) were poor inhibitors, the triketones (e.g., **12**–**15**) were more potent (Figure 3 B). However, the phenolic triketone “mimic” 2′,6′‐dihydroxyacetophenone **16** was a relatively poor OGFOD1 inhibitor. The most potent template identified for OGFOD1 inhibition was manifested in the C‐5 substituted barbiturate derivatives (**18**–**20**); such compounds are readily enolized, which is likely beneficial for metal chelation. *N*‐Methylation of the barbiturate ring (e.g., **20**) improved potency, as did the introduction of an acyl substituent on C‐5 of the barbiturate core (e.g., **18** compared to **17**). Replacement of the C‐2 oxygen with sulfur (thiobarbituric acid **19**) did not substantially increase the potency relative to the oxygen analogue (i.e., **18**), while replacement of the oxygen at C‐4 with a nitrogen (e.g., **21**) abolished any inhibitory activity (Figure 3 B). We therefore focused on obtaining selective OGFOD1 inhibitors by modifying the C‐5 position of a 1,3‐dimethyl barbiturate core. Notably, compounds **22** and **23**, which combine a barbiturate core with a glycinamide ethyl ester side chain, did not manifest clear selectivity for OGFOD1 over PHD2 (Figure 4; Figure S6).

Therefore, **24** was prepared, in which structural features of **22** are “merged” with HPPD/plant growth inhibitors **1**–**3**, by introducing a C‐5 aromatic acyl substituent in place of a glycinamide. To investigate the optimal spacing between the barbiturate and the aromatic side chain, the initial screen encompassed a 1,3‐dimethyl barbiturate core bearing acetyl **20**, benzoyl **24**, phenylacetyl **25**, and hydrocinnamoyl **26** (or **CCT3**) substituents. The resulting aromatic compounds potently inhibited OGFOD1, with an increase in potency being observed upon extending the carbon chain length from **24** (IC_50_=30 μm) to **25** (IC_50_=2 μm), and from **25** to **26** (**CCT3**) (IC_50_=0.7 μm; assays performed using 1 μm OGFOD1; Figure S7). Extending the side chain further (e.g., **27**, **28**) did not provide a substantial increase in potency. Importantly, this series displayed no observable PHD2 inhibition at concentrations up to 100 μm (Figure S8). These results suggest that the C‐5 substituent is important for obtaining selectivity between OGFOD1 and PHD2.

The importance of the *N*‐alkyl substituents for inhibition was examined with barbiturates bearing C‐5 cyclopentyl substituents (Figure 4 C). Analogues bearing *N*,*N′*‐dimethyl (**29**), *N*,*N′*‐diethyl (**30**), and *N*,*N′*‐dicyclohexyl (**31**) groups were prepared (Figure 4 C); analogues **30** and **31** were less potent than **29**, suggesting that substituents larger than methyl groups may not be favorable for OGFOD1 inhibition (Figure S9). Note, however, that GSK1278863 **6** potently inhibits OGFOD1 despite the presence of *N*,*N′*‐dicyclohexyl groups (Figure 2 A).^33^

Modeling suggests that the aryl side chains of **25** and **CCT3** likely do not fit in the OGFOD1 2OG binding site due to steric constraints; instead, the aryl ring may bind in the substrate binding site, potentially contributing to the selectivity of these inhibitors. Varying the C‐5 side chain with different mono‐ and bicyclic aromatic and saturated substituents (**32**–**45**) did not have a substantial impact on potency (Figure 4 D, Figure S10). Similarly, changing the nature of the C‐5 link from a ketone to an amide (i.e., **46**), or extending the carbon chain length beyond two carbons between the carbonyl and the substituent had little effect (Figure 4D).

**Figure 5:**
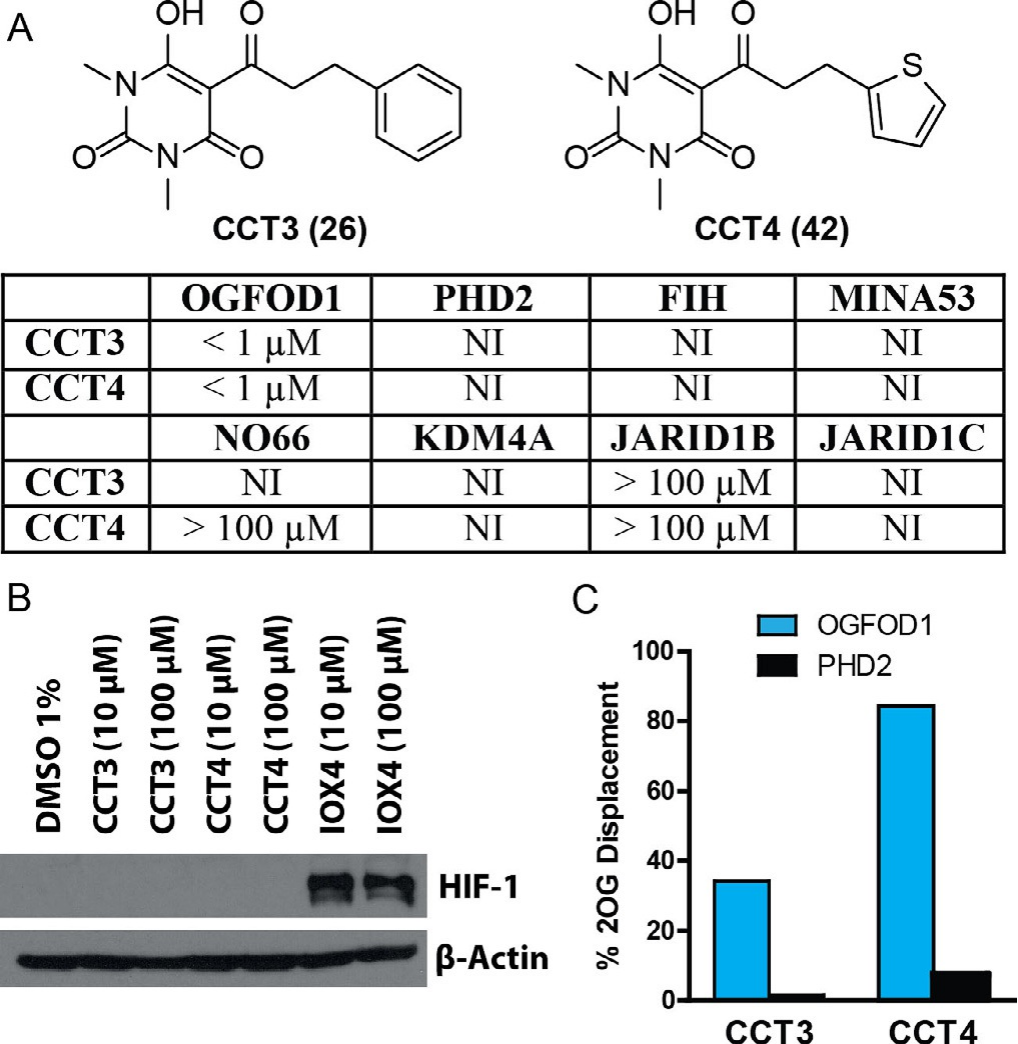
Selectivity of lead compounds against 2OG oxygenases: (A) Comparison of inhibition data for compounds **CCT3** and **CCT4** against OGFOD1 and other human 2OG oxygenases. NI: no inhibition observed. (B) Western blot (antibody specific for HIFα) showing the lack of impact of **CCT3** and **CCT4** on the activity of PHD enzymes in HeLa cells.^33^ In comparison, the known PHD inhibitor IOX4 inhibits the hydroxylation of HIFα, preventing proteasomal degradation.^34^ (C) Extent of displacement of 2OG from the active site of OGFOD1 (blue) and PHD2 (black) by **CCT3** and **CCT4** as observed by CPMG‐edited ^1^H‐NMR. See Figure S15 (Supporting Information) for additional details.

On the basis of these SAR studies, in particular those examining the impact of the C‐5 and barbiturate *N*‐alkyl substituents, the sub‐micromolar potency inhibitors **CCT3** (**26**) and **CCT4** (**42**) were selected for further characterization (Figure 4 and Figure S11). These two inhibitors were screened for inhibitory activity against a panel of human 2OG oxygenases (Figure 5 A). Of the human enzymes screened, including the HIF prolyl hydroxylase PHD2, the ribosomal oxygenases MINA53 and NO66 (which hydroxylate histidinyl residues in ribosomal proteins)^20^ and the asparaginyl hydroxylase factor‐inhibiting HIF (FIH), **CCT3** and **CCT4** only potently inhibited OGFOD1 (with IC_50_ values of 0.73 and 0.69 μm, respectively; Figures S7, S11). Additionally, these compounds showed poor, or no, inhibition of more distantly related 2OG oxygenases, such as the histone demethylases KDM4A, JARID1B, and JARID1C (Figure 5 A). **CCT3** and **CCT4** did potently inhibit the structurally close yeast OGFOD1 homologues Tpa1p and Ofd1 (which hydroxylate RPS23 prolyl residues; Figure S12), consistent with the high levels of similarity between the homologous active sites.^10^ It is also notable that the sensitivity of the current hydroxylation assay is a limitation for ranking the activities of the potent inhibitors, as the IC_50_ values obtained are close to the OGFOD1 concentration used (i.e., the minimum IC_50_ value that can be measured is 0.5 μm); thus, these inhibitors may be more potent than represented by the currently reported IC_50_ values.

To validate the in vitro inhibition results, direct binding interactions between inhibitors **CCT3** and **CCT4** and OGFOD1 and PHD2 were assessed by NMR analysis. While both inhibitors were observed to strongly bind to OGFOD1, as observed using ^1^H Carr‐Purcell‐Meiboom‐Gill (CPMG) analyses (Figure S13), only weak binding to PHD2 was observed by water‐Ligand Observe Gradient Spectroscopy (wLOGSY) experiments (Figure S14). Competition experiments between the inhibitors and enzyme‐bound 2OG were conducted by monitoring the recovery of the enzyme‐free 2OG methylene peak at 2.35 ppm using CPMG‐edited ^1^H‐NMR upon addition of the inhibitor.^35^ The results indicate that **CCT3** and **CCT4** are capable of displacing bound 2OG from the active site of OGFOD1, but not from that of PHD2 (Figure 5 C, S15).

We examined the potential inhibition of PHD enzymes by **CCT3** and **CCT4** using the HeLa human cell line.^36^ Compared to the known PHD inhibitor IOX4,^34^ **CCT3** and **CCT4** did not stabilize HIFα (Figure 5 B). PHD‐catalyzed hydroxylation targets HIFα for proteasomal degradation, indicating that these compounds do not inhibit the PHDs in cells. Based on a MDR1‐MDCK assay (performed by Cyprotex, UK; see the Supporting Information), **CCT3** and **CCT4** demonstrate good cell permeability properties, and are predicted to be permeable to the blood brain barrier. Liver microsome stability studies indicate only low levels of clearance of **CCT3** and **CCT4** (Cyprotex, UK).

## Discussion

Our results demonstrate the viability of a template‐based approach for the development of selective 2OG oxygenase/prolyl hydroxylase inhibitors capable of differentiating between closely related active sites, such as those of the human prolyl hydroxylases OGFOD1 and PHD2. Furthermore, the optimized inhibitors also did not inhibit the other 2OG oxygenases tested, including other ribosomal oxygenases, as well as histone demethylases. The results suggest that specific inhibitor ‘templates’ may be preferred for certain oxygenases or oxygenase subfamilies, as supported by work implying differential selectivity between PH and JmjC histone demethylase inhibitors.^37^ This preference for particular templates may even extend to enzymes with closely related active sites. Appropriately modified barbiturate‐based inhibitors may selectively inhibit OGFOD1 because of their ability to support substituents, which extend towards active‐site residues present in OGFOD1 but not in PHD2. By contrast, the glycinamide side chain present in many PHD2 inhibitors (including several compounds in clinical trials, e.g., **6** and **7**) is clearly not required for potent OGFOD1 inhibition. It should also be noted that potent PHD2 inhibitors without a glycinamide side chain are known.^33^, ^34^ In future work, it will be of interest to further explore the selectivity of the compounds reported here. In this regard, studies with the procollagen C‐4 (and C‐3) prolyl hydroxylases are of particular interest, especially as the procollagen C‐4 PHs are potential therapeutic targets.^38^

There is considerable academic and pharmaceutical interest in developing chemical probe compounds to investigate the biological functions of 2OG oxygenases.^39^ Our results suggest that the development of leads based on known pharmaceutical and agrochemical ‘templates’ (some of which can penetrate the blood–brain barrier) with well‐studied physicochemical properties, such as barbiturates, will be a productive strategy. The combination of the tricarbonyl barbiturate template of the PHD2 inhibitor GSK1278863 **6** with the side chains of agrochemical oxygenase inhibitors (e.g., prohexadione‐calcium), followed by subsequent optimization, yielded potent and selective OGFOD1 inhibitors. Future work can now be focused on applying these OGFOD1 inhibitors to investigate the biological roles of OGFOD1, and applying a similar inhibitor development strategy to other ribosomal oxygenases. Based on what is observed in these functional studies, further optimization of these inhibitors may be warranted (e.g., if penetration of the blood–brain barrier is desirable).

It is important to note that many PHD2 inhibitors reported in the literature, including those screened in this work, and those currently in clinical trials, also inhibit OGFOD1.^33^ Indeed, they may also inhibit other human prolyl hydroxylases and 2OG oxygenases, including those for which assays are currently not available.^10^, ^40^ From a clinical perspective, it is also important to note that the barbiturate‐related ′triketone′ HPPD inhibitor nitisinone, which is used in the treatment of type I tyrosinaemia,^27^ is an OGFOD1 inhibitor, something that might be taken into consideration if nitisinone successors with improved properties are pursued. In the present work, we have demonstrated that it is possible to attain selectivity between different 2OG oxygenases, with lead compounds that inhibit OGFOD1, but not PHD2. Such “biochemical selectivity” is not necessarily an issue with clinical applications as the desired pharmacological effect/safety profile may be achieved by controlling metabolism and tissue distribution. However, we propose that, at least for chronic applications, biochemical selectivity could and should be optimized during the development of 2OG oxygenase inhibitors. We also hope that inhibitors selective for particular 2OG oxygenases may help enable their individual biological roles to be deciphered.

## Conflict of interest

The authors declare no conflict of interest.

## Supporting information

As a service to our authors and readers, this journal provides supporting information supplied by the authors. Such materials are peer reviewed and may be re‐organized for online delivery, but are not copy‐edited or typeset. Technical support issues arising from supporting information (other than missing files) should be addressed to the authors.

Supplementary

## References

[1] C. J. Schofield , P. J. Ratcliffe , Nat. Rev. Mol. Cell Biol. 2004, 5, 343–354. 15122348 10.1038/nrm1366

[2] S. E. Wilkins , M. I. Abboud , R. L. Hancock , C. J. Schofield , ChemMedChem 2016, 11, 773–786. 26997519 10.1002/cmdc.201600012 PMC4848768

[3] J. D. Webb , M. L. Coleman , C. W. Pugh , Cell. Mol. Life Sci. 2009, 66, 3539–3554. 19756382 10.1007/s00018-009-0147-7 PMC11115642

[4] W. S. Aik , R. Chowdhury , I. J. Clifton , R. J. Hopkinson , T. Leissing , M. A. McDonough , R. Nowak , C. J. Schofield , L. J. Walport , 2-Oxoglutarate-Dependent Oxygenases, Royal Society of Chemistry, Cambridge, 2015, p. 59–94.

[5] C. Loenarz , C. J. Schofield , Nat. Chem. Biol. 2008, 4, 152–156. 18277970 10.1038/nchembio0308-152

[6] K. L. Gorres , R. T. Raines , Crit. Rev. Biochem. Mol. Biol. 2010, 45, 106–124. 20199358 10.3109/10409231003627991 PMC2841224

[7] R. S. Singleton , P. Liu-Yi , F. Formenti , W. Ge , R. Sekirnik , R. Fischer , J. Adam , P. J. Pollard , A. Wolf , A. Thalhammer , C. Loenarz , E. Flashman , A. Yamamoto , M. L. Coleman , B. M. Kessler , P. Wappner , C. J. Schofield , P. J. Ratcliffe , M. E. Cockman , Proc. Natl. Acad. Sci. USA 2014, 111, 4031–4036. 24550447 10.1073/pnas.1314482111 PMC3964040

[8] C. Loenarz , R. Sekirnik , A. Thalhammer , W. Ge , E. Spivakovsky , M. M. Mackeen , M. A. McDonough , M. E. Cockman , B. M. Kessler , P. J. Ratcliffe , A. Wolf , C. J. Schofield , Proc. Natl. Acad. Sci. USA 2014, 111, 4019–4024. 24550462 10.1073/pnas.1311750111 PMC3964080

[9] M. J. Katz , J. M. Acevedo , C. Loenarz , D. Galagovsky , P. Liu-Yi , M. Pérez-Pepe , A. Thalhammer , R. Sekirnik , W. Ge , M. Melani , M. G. Thomas , S. Simonetta , G. L. Boccaccio , C. J. Schofield , M. E. Cockman , P. J. Ratcliffe , P. Wappner , Proc. Natl. Acad. Sci. USA 2014, 111, 4025–4030. 24550463 10.1073/pnas.1314485111 PMC3964085

[10] S. Horita , J. S. Scotti , C. Thinnes , Y. S. Mottaghi-Taromsari , A. Thalhammer , W. Ge , W. Aik , C. Loenarz , C. J. Schofield , M. A. McDonough , Structure 2015, 23, 639–652. 25728928 10.1016/j.str.2015.01.014 PMC4396695

[11] T. M. Schmeing , V. Ramakrishnan , Nature 2009, 461, 1234–1242. 19838167 10.1038/nature08403

[12] W. V. Gilbert , Trends Biochem. Sci. 2011, 36, 127–132. 21242088 10.1016/j.tibs.2010.12.002 PMC3056915

[13] M. V. Nesterchuk , P. V. Sergiev , O. A. Dontsova , Acta Naturae 2011, 3, 22–33. 22649682 PMC3347575

[14] L. E. Alksne , R. A. Anthony , S. W. Liebman , J. R. Warner , Proc. Natl. Acad. Sci. USA 1993, 90, 9538–9541. 8415737 10.1073/pnas.90.20.9538 PMC47604

[15] R. Chowdhury , I. K. Leung , Y. M. Tian , M. I. Abboud , W. Ge , C. Domene , F. X. Cantrelle , I. Landrieu , A. P. Hardy , C. W. Pugh , P. J. Ratcliffe , T. D. Claridge , C. J. Schofield , Nat. Commun. 2016, 7, 12673. 27561929 10.1038/ncomms12673 PMC5007464

[16] N. R. Rose , M. A. McDonough , O. N. King , A. Kawamura , C. J. Schofield , Chem. Soc. Rev. 2011, 40, 4364–4397. 21390379 10.1039/c0cs00203h

[17] J. D. Vasta , K. A. Andersen , K. M. Deck , C. P. Nizzi , R. S. Eisenstein , R. T. Raines , ACS Chem. Biol. 2016, 11, 193–199. 26535807 10.1021/acschembio.5b00749 PMC4798942

[18] M. C. Chan , J. P. Holt-Martyn , C. J. Schofield , P. J. Ratcliffe , Mol. Aspects Med. 2016, 47–48, 54–75. 10.1016/j.mam.2016.01.001 26791432

[19] K. M. Keeling , X. Xue , G. Gunn , D. M. Bedwell , Annu. Rev. Genomics Hum. Genet. 2014, 15, 371–394. 24773318 10.1146/annurev-genom-091212-153527 PMC5304456

[20] W. Ge , A. Wolf , T. Feng , C. H. Ho , R. Sekirnik , A. Zayer , N. Granatino , M. E. Cockman , C. Loenarz , N. D. Loik , A. P. Hardy , T. D. Claridge , R. B. Hamed , R. Chowdhury , L. Gong , C. V. Robinson , D. C. Trudgian , M. Jiang , M. M. Mackeen , J. S. McCullagh , Y. Gordiyenko , A. Thalhammer , A. Yamamoto , M. Yang , P. Liu-Yi , Z. Zhang , M. Schmidt-Zachmann , B. M. Kessler , P. J. Ratcliffe , G. M. Preston , M. L. Coleman , C. J. Schofield , Nat. Chem. Biol. 2012, 8, 960–962. 23103944 10.1038/nchembio.1093 PMC4972389

[21] R. Chowdhury , R. Sekirnik , N. C. Brissett , T. Krojer , C. H. Ho , S. S. Ng , I. J. Clifton , W. Ge , N. J. Kershaw , G. C. Fox , J. R. C. Muniz , M. Vollmar , C. Phillips , E. S. Pilka , K. L. Kavanagh , F. von Delft , U. Oppermann , M. A. McDonough , A. J. Doherty , C. J. Schofield , Nature 2014, 510, 422–426. 24814345 10.1038/nature13263 PMC4066111

[22] S. Markolovic , Q. Zhuang , S. E. Wilkins , C. D. Eaton , M. I. Abboud , M. J. Katz , H. E. McNeil , R. K. Leśniak , C. Hall , W. B. Struwe , R. Konietzny , S. Davis , M. Yang , W. Ge , J. L. P. Benesch , B. M. Kessler , P. J. Ratcliffe , M. E. Cockman , R. Fischer , P. Wappner , R. Chowdhury , M. L. Coleman , C. J. Schofield , Nat. Chem. Biol. 2018, 14, 688–695. 29915238 10.1038/s41589-018-0071-y PMC6027965

[23] T. Feng , A. Yamamoto , S. E. Wilkins , E. Sokolova , L. A. Yates , M. Münzel , P. Singh , R. J. Hopkinson , R. Fischer , M. E. Cockman , J. Shelley , D. C. Trudgian , J. Schödel , J. S. McCullagh , W. Ge , B. M. Kessler , R. J. Gilbert , L. Y. Frolova , E. Alkalaeva , P. J. Ratcliffe , C. J. Schofield , M. L. Coleman , Mol. Cell 2014, 53, 645–654. 24486019 10.1016/j.molcel.2013.12.028 PMC3991326

[24] M. A. McDonough , C. Loenarz , R. Chowdhury , I. J. Clifton , C. J. Schofield , Curr. Opin. Struct. Biol. 2010, 20, 659–672. 20888218 10.1016/j.sbi.2010.08.006

[25] R. Chowdhury , M. A. McDonough , J. Mecinović , C. Loenarz , E. Flashman , K. S. Hewitson , C. Domene , C. J. Schofield , Structure 2009, 17, 981–989. 19604478 10.1016/j.str.2009.06.002

[26] C. C. Thinnes , A. Tumber , C. Yapp , G. Scozzafava , T. Yeh , M. C. Chan , T. A. Tran , K. Hsu , H. Tarhonskaya , L. J. Walport , S. E. Wilkins , E. D. Martinez , S. Müller , C. W. Pugh , P. J. Ratcliffe , P. E. Brennan , A. Kawamura , C. J. Schofield , Chem. Commun. 2015, 51, 15458–15461. 10.1039/c5cc06095h 26345662

[27] P. J. McKiernan , Drugs 2006, 66, 743–750. 16706549 10.2165/00003495-200666060-00002

[28] G. R. Moran , Arch. Biochem. Biophys. 2005, 433, 117–128. 15581571 10.1016/j.abb.2004.08.015

[29] M. J. Koury , V. H. Haase , Nat. Rev. Nephrol. 2015, 11, 394. 26055355 10.1038/nrneph.2015.82 PMC4497972

[30] R. Chowdhury , J. I. Candela-Lena , M. C. Chan , D. J. Greenald , K. K. Yeoh , Y. M. Tian , M. A. McDonough , A. Tumber , N. R. Rose , A. Conejo-Garcia , M. Demetriades , S. Mathavan , A. Kawamura , M. K. Lee , F. van Eeden , C. W. Pugh , P. J. Ratcliffe , C. J. Schofield , ACS Chem. Biol. 2013, 8, 1488–1496. 23683440 10.1021/cb400088q

[31] R. Chowdhury , K. K. Yeoh , Y. M. Tian , L. Hillringhaus , E. A. Bagg , N. R. Rose , I. K. Leung , X. S. Li , E. C. Woon , M. Yang , M. A. McDonough , O. N. King , I. J. Clifton , R. J. Klose , T. D. Claridge , P. J. Ratcliffe , C. J. Schofield , A. Kawamura , EMBO Rep. 2011, 12, 463–469. 21460794 10.1038/embor.2011.43 PMC3090014

[32] F. H. Niesen , H. Berglund , M. Vedadi , Nat. Protoc. 2007, 2, 2212–2221. 17853878 10.1038/nprot.2007.321

[33] T.-L. Yeh , T. M. Leissing , M. I. Abboud , C. C. Thinnes , O. Atasoylu , J. P. Holt-Martyn , D. Zhang , A. Tumber , K. Lippl , C. T. Lohans , I. K. H. Leung , H. Morcrette , I. J. Clifton , T. D. W. Claridge , A. Kawamura , E. Flashman , X. Lu , P. J. Ratcliffe , R. Chowdhury , C. W. Pugh , C. J. Schofield , Chem. Sci. 2017, 8, 7651–7668. 29435217 10.1039/c7sc02103h PMC5802278

[34] M. C. Chan , O. Atasoylu , E. Hodson , A. Tumber , I. K. Leung , R. Chowdhury , V. Gómez-Pérez , M. Demetriades , A. M. Rydzik , J. Holt-Martyn , Y. M. Tian , T. Bishop , T. D. Claridge , A. Kawamura , C. W. Pugh , P. J. Ratcliffe , C. J. Schofield , PLoS One 2015, 10, e0132004. 10.1371/journal.pone.0132004 PMC4492579 26147748

[35] M. I. Abboud , T. E. McAllister , I. K. H. Leung , R. Chowdhury , C. Jorgensen , C. Domene , J. Mecinović , K. Lippl , R. L. Hancock , R. J. Hopkinson , A. Kawamura , T. D. W. Claridge , C. J. Schofield , Chem. Commun 2018, 54, 3130–3133. 10.1039/c8cc00387d PMC5885369 29522057

[36] Y. M. Tian , K. K. Yeoh , M. K. Lee , T. Eriksson , B. M. Kessler , H. B. Kramer , M. J. Edelmann , C. Willam , C. W. Pugh , C. J. Schofield , P. J. Ratcliffe , J. Biol. Chem. 2011, 286, 13041–13051. 21335549 10.1074/jbc.M110.211110 PMC3075650

[37] C. C. Thinnes , K. S. England , A. Kawamura , R. Chowdhury , C. J. Schofield , R. J. Hopkinson , Biochim. Biophys. Acta Gene Regul. Mech. 2014, 1839, 1416–1432. 10.1016/j.bbagrm.2014.05.009 PMC4316176 24859458

[38] J. D. Vasta , R. T. Raines , J Med Chem. 2018, 61, 10403–10411. 29986141 10.1021/acs.jmedchem.8b00822 PMC6344319

[39] C. H. Arrowsmith , J. E. Audia , C. Austin , J. Baell , J. Bennett , J. Blagg , C. Bountra , P. E. Brennan , P. J. Brown , M. E. Bunnage , C. Buser-Doepner , R. M. Campbell , A. J. Carter , P. Cohen , R. A. Copeland , B. Cravatt , J. L. Dahlin , D. Dhanak , A. M. Edwards , M. Frederiksen , S. V. Frye , N. Gray , C. E. Grimshaw , D. Hepworth , T. Howe , K. V. Huber , J. Jin , S. Knapp , J. D. Kotz , R. G. Kruger , D. Lowe , M. M. Mader , B. Marsden , A. Mueller-Fahrnow , S. Müller , R. C. O'Hagan , J. P. Overington , D. R. Owen , S. H. Rosenberg , B. Roth , B. Roth , R. Ross , M. Schapira , S. L. Schreiber , B. Shoichet , M. Sundström , G. Superti-Furga , J. Taunton , L. Toledo-Sherman , C. Walpole , M. A. Walters , T. M. Willson , P. Workman , R. N. Young , W. J. Zuercher , Nat. Chem. Biol. 2015, 11, 536–541. 26196764 10.1038/nchembio.1867 PMC4706458

[40] F. McMurray , M. Demetriades , W. Aik , M. Merkestein , H. Kramer , D. S. Andrew , C. L. Scudamore , T. A. Hough , S. Wells , F. M. Ashcroft , M. A. McDonough , C. J. Schofield , R. D. Cox , PLoS One 2015, 10, e0121829. 10.1371/journal.pone.0121829 PMC4382163 25830347

