# Supplementary material for: Selective Inhibitors of a Human Prolyl Hydroxylase (OGFOD1) Involved in Ribosomal Decoding
Source: Chemistry. 2019 Jan 8;25(8):2019–24. doi: 10.1002/chem.201804790 (PMC6471485; doi:10.1002/chem.201804790)
Supplement: Supplementary file 1 — Supplementary [file CHEM-25-2019-s001.pdf]

CHEMISTRY
A European Journal

Supporting Information

Selective Inhibitors of a Human Prolyl Hydroxylase (OGFOD1) Involved in Ribosomal Decoding

Cyrille C. Thinnes,[a] Christopher T. Lohans,[a] Martine I. Abboud,[a] Tzu-Lan Yeh,[a] Anthony Tumber,[a, b] Radosław P. Nowak,[b, c] Martin Attwood,[d] Matthew E. Cockman,[d] Udo Oppermann,[b] Christoph Loenarz,[a, e] and Christopher J. Schofield*[a]

chem_201804790_sm_miscellaneous_information.pdf

Author Contributions

C.T. Conceptualization: Equal; Investigation: Lead; Writing – original draft: Equal
C.L. Investigation: Supporting; Writing – original draft: Equal; Writing – review & editing: Lead
M.A. Investigation: Supporting; Writing – review & editing: Supporting
T.Y. Investigation: Supporting
A.T. Investigation: Supporting; Writing – original draft: Supporting
R.N. Investigation: Supporting; Writing – review & editing: Supporting
M.A. Investigation: Supporting
M.C. Supervision: Supporting
U.O. Supervision: Supporting
C.L. Funding acquisition: Lead; Supervision: Supporting; Writing – review & editing: Supporting
C.S. Funding acquisition: Lead; Project administration: Lead; Supervision: Lead; Writing – original draft: Equal; Writing – review & editing: Equal.

Supporting Information Contents

Experimental Section	S2
Impact of PHD inhibitors on OGFOD1 and PHD2 activity	S25
Impact of HPPD and plant growth inhibitors on OGFOD1 and PHD2 activity	S26
Stabilization of OGFOD1 and PHD2 by HPPD and plant growth inhibitors	S27
Stabilization of OGFOD1 and PHD2 by PHD inhibitors	S28
Predicted metal coordination of GSK1278863 and sulcotrione	S29
Inhibition of PHD2 and OGFOD2 by **23**	S30
Determination of IC_{50} values for **24**, **25**, and **CCT3** against OGFOD1	S31
Inhibition of PHD2 by triketone derivatives	S32
Impact of barbiturate *N*,*N'*-alkyl groups on OGFOD1 inhibition	S33
Inhibition of OGFOD1 by C-5 substituted barbiturate derivatives	S34
Determination of IC_{50} values for **CCT4** against OGFOD1	S35
Determination of IC_{50} values for **CCT3** and **CCT4** against Tpa1p and Ofd1	S36
CPMG NMR analyses of **CCT3** and **CCT4** binding to OGFOD1	S37
wLOGSY NMR analyses of **CCT3** and **CCT4** binding with OGFOD1 and PHD2	S38
2OG displacement experiments by CPMG NMR	S39
ADMET assays for **CCT3** and **CCT4**	S40
References	S41

Experimental Section

General

Unless noted otherwise, all reagents were from Sigma-Aldrich and were used as supplied. When required, solvents were dried according to reported procedures.[1] Water was purified and filtered using a Millipore MilliQ system. Moisture-sensitive reagents were handled under a nitrogen atmosphere using flame-dried glassware. Aluminium plates coated with 60 F254 silica were used for thin layer chromatography, and visualized at 254 nm. Reactions were purified by flash column chromatography using either a glass column packed with Kieselgel 60 silica or a Biotage SP4 chromatography system.

Nuclear magnetic resonance (NMR) spectra for products were acquired on Bruker Avance spectrometers using standard parameters. Samples were prepared in deuterated solvents as indicated. A Bruker Tensor 27 FT-IR spectrometer was used for acquiring infrared (IR) spectra. Melting points were acquired using a Gallenkamp Hot Stage apparatus. Mass spectra were acquired with VG MassLab 20-250, Micromass Platform 1, Bruker MicroTOF, and Micromass GCT spectrometers.

Synthetic Procedures

5-Acetyl-6-hydroxypyrimidine-2,4(1H,3H)-dione (18)[2]

Barbituric acid (1.3 g, 10 mmol) was heated at reflux in acetic anhydride (30 mL) in the presence of 3 drops of conc. sulphuric acid for 60 min. The reaction mixture was concentrated to a total volume of about 15 mL under reduced pressure and then cooled in an ice bath. The resulting precipitate was collected by filtration and washed with hot water and acetone before being dried under reduced pressure to give **18** (872 mg, 51 %) as a light-brown powder.

mp 309 °C; δ_H (400 MHz, DMSO-d_6) 11.80 (1 H, br. s., N*H*), 11.07 (1 H, br. s., N*H*), 2.59 (3 H, s, CH_3); δ_C (100 MHz, DMSO-d_6) 195.4 (*C*=O), 172.2 (*C*=O), 162.8 (*C*=O),149.5 (*C*=O), 95.9 (C_5), 24.3 (CH_3); ν_{max}/cm^{-1} 1735 (ketone C=O), 1627 (amide C=O); *m*/*z* (ESI$^-$) 169 ([M-H]$^-$); HRMS (ESI$^-$) $C_6H_5O_4N_2$, ([M-H]$^-$) calcd 169.0255; found 169.0253.

5-Acetyl-6-hydroxy-2-thioxo-2,3-dihydropyrimidin-4(1H)-one (19)

Thiobarbituric acid (1.4 g, 10 mmol) was heated at reflux in acetic anhydride (30 mL) in the presence of 3 drops of conc. sulphuric acid for 60 min. The reaction mixture was concentrated to a total volume of about 15 mL under reduced pressure and then cooled in an ice bath. The resulting precipitate was collected by filtration and washed with hot water and acetone before being dried under reduced pressure to give **19** (560 mg, 30 %) as a dark-brown powder.

mp (decomposition) > 200 °C; δ_H (400 MHz, DMSO-d_6) 2.60 (3 H, s, CH_3); δ_C (100 MHz, DMSO-d_6) 197.0 (*C*=O), 177.2 (*C*=O), 97.6 (C_5), 24.8 (CH_3); ν_{max}/cm^{-1} 1722 (ketone C=O), 1655 (amide C=O); *m*/*z* (FI) 186 ([M]$^+$); HRMS (FI) $C_6H_6N_2O_3S$, ([M]$^+$) calcd 186.0099; found 186.0094.

5-Acetyl-6-hydroxy-1,3-dimethylpyrimidine-2,4(1H,3H)-dione (20)

1,3-Dimethylbarbituric acid (1.6 g, 10 mmol) was heated at reflux in acetic anhydride (30 mL) in the presence of 3 drops of sulphuric acid for 60 min. The reaction mixture was concentrated to a total volume of about 15 mL under reduced pressure and then cooled in an ice bath. The resulting precipitate was collected by filtration and washed with hot water and acetone before being dried under reduced pressure to give **20** (1.68 g, 85 %) an off-white powder.

mp 104 °C; δ_H (400 MHz, DMSO-d_6) 3.19 (6 H, s, NCH_3), 2.64 (3 H, s, CH_3); δ_C (100 MHz, DMSO-d_6) 195.0 (*C*=O), 150.5 (*C*=O), 96.4 (C_5), 28.1 (NCH_3), 24.5 (CH_3); ν_{max}/cm^{-1} 1722

(ketone C=O), 1655 (amide C=O); *m/z* (FI) 198 ($[M]^+$); HRMS (FI) $C_8H_{10}N_2O_4$, ($[M]^+$) calcd 198.0641; found 198.0643.

6-Amino-1,3-dimethylpyrimidine-2,4(1H,3H)-dione (S1)[3]

A solution of *N*,*N*'-dimethylurea (3.52 g, 40 mmol) and cyanoacetic acid (3.74 g, 44 mmol) in acetic anhydride (30 mL) was stirred at 70 °C for 126 h. The solvent was evaporated *in vacuo* and the resulting oily residue was diluted with NaOH (5 M in H_2O, 15 mL). The precipitate was collected by filtration, washed with cold H_2O, and purified by recrystallisation from a 1:1 mixture of MeOH and H_2O to give **S1** (3.5 g, 57 %) as a white powder, which was used directly in the next step.

δ_H (400 MHz, DMSO-d_6) 4.71 (1 H, s, C*H*), 3.24 (3 H, s, NCH_3), 3.07 (3 H, s, NCH_3); δ_C (100 MHz, DMSO-d_6) 162.0, 155.4, 152.1, 75.4, 29.7, 27.5; *m/z* (ESI^-) 154 ($[M-H]^-$).

5-Acetyl-6-amino-1,3-dimethylpyrimidine-2,4(1H,3H)-dione (21)

S1 (1.6 g, 10 mmol) was heated at reflux in acetic anhydride (30 mL) in the presence of 3 drops of sulphuric acid for 60 min. The reaction mixture was concentrated to a total volume of about 15 mL under reduced pressure and then cooled in an ice bath. The resulting precipitate was collected by filtration and washed with hot water and acetone before being dried under reduced pressure to give **21** (614 mg, 31 %) as a light-brown powder.

mp 265 °C; δ_H (400 MHz, pyridine-d_5) 5.14 (6 H, s, NCH_3), 4.93 (3 H, s, COCH_3); δ_C (100 MHz, pyridine-d_5) 161.0 (*C*=O), 160.9 (*C*=O), 152.9, 105.5 (C_5), 29.9 (N*C*H_3), 28.2 (N*C*H_3), 18.2 (CO*C*H_3); ν_{max}/cm^{-1} 1701 (ketone C=O), 1660 (C=O); no mass spectrum could be obtained.

Ethyl (6-Hydroxy-2,4-dioxo-1,2,3,4-tetrahydropyrimidine-5-carbonyl)glycinate (22)

Ethyl isocyanatoacetate (560 μL, 5 mmol) was slowly added to a stirred solution of barbituric acid (512 mg, 4 mmol) in diisopropylethylamine (2 mL), CH_2Cl_2 (3 mL) and DMF (5 mL) at room temperature. The resulting solution was stirred at room temperature for 16 h after which the solvent was evaporated under reduced pressure. The residue was redissolved in a mixture of EtOH (5 mL) and NaOH (1 M in H_2O) and the resulting mixture was stirred at room temperature for 60 min. The pH was adjusted to 7 with HCl (1 M in H_2O) and the resulting solid was collected by filtration, washed with H_2O, and dried under vacuum to give **22** (812 mg, 79 %) as a white powder.

mp >250 °C; δ_H (400 MHz, DMSO-d_6) 3.96 - 4.19 (4 H, m, O$CH_2$$CH_3$ and NCH_2), 1.21 (1 H, t, J=7.0 Hz, OCH_2CH_3); δ_C (100 MHz, DMSO-d_6) 170.4 (*C*=O), 169.6 (*C*=O), 150.2, 61.0 (O$CH_2$$CH_3$), 41.1 (N$CH_2$), 29.9 (N$CH_3$), 14.6 ($OCH_2CH_3$); ν_{max}/cm^{-1} 1718 (C=O), 1650 (C=O), 1620 (C=O); *m/z* (ESI$^-$) 256 ([M-H]$^-$); HRMS (ESI$^-$) $C_9H_{11}O_6N_3$, ([M-H]$^-$) calcd 256.0575; found 256.0580.

Ethyl (6-hydroxy-1,3-dimethyl-2,4-dioxo-1,2,3,4-tetrahydropyrimidine-5-carbonyl)glycinate (23)

Ethyl isocyanatoacetate (560 μL, 5 mmol) was slowly added to a stirred solution of 1,3-dimethylbarbituric acid (624 mg, 4 mmol) in diisopropylethylamine (2 mL), CH_2Cl_2 (3 mL) and DMF (5 mL) at room temperature. The resulting solution was stirred at room temperature for 16 h, and then the solvent was evaporated under reduced pressure. The residue was redissolved in a mixture of EtOH (5 mL) and NaOH (1 M in H_2O) and the resulting mixture was stirred at room temperature for 60 min. The pH was adjusted to 7 with HCl (1 M in H_2O)

and the resulting solid was collected by filtration, washed with H_2O, and dried under *vacuum* to give **23** (821 mg, 72 %) as a white powder.

mp > 250 °C; δ_H (400 MHz, DMSO-d_6) 4.12 (2 H, q, J=7.0 Hz, OCH_2CH$_3$), 4.07 (2 H, d, J=5.5 Hz, NHCH_2), 3.16 (6 H, s, NCH_3), 1.21 (3 H, t, J=7.0 Hz, OCH$_2$$CH_3$); δ_C (100 MHz, DMSO-d_6) 170.4 (*C*=O), 169.6 (*C*=O), 151.3, 61.0 (O*C*H$_2$CH$_3$), 41.3 (N*C*H$_2$), 27.7 (N*C*H$_3$), 14.6 (OCH$_2$*C*H$_3$); ν_{max}/cm^{-1} 1756 (C=O), 1737 (C=O), 1724 (C=O), 1671 (C=O); *m*/*z* (ESI$^-$) 284 ([M-H]$^-$); HRMS (ESI$^-$) $C_{11}H_{14}O_6N_3$, ([M-H]$^-$) calcd 284.0877; found 284.0889.

5-Benzoyl-6-hydroxy-1,3-dimethylpyrimidine-2,4(1H,3H)-dione (24)[4]

Benzoyl chloride (1.2 mL, 10 mmol) was slowly added to a stirred solution of 1,3-dimethylbarbituric acid (1.6 g, 10 mmol) in pyridine (6 mL) at room temperature. The resulting reaction mixture was stirred for an additional 2 h at room temperature before being slowly added to a stirred mixture of methanol (6 mL), H_2O (5 mL), and concentrated HCl aq. (15 mL). The resulting suspension was stirred for an additional 30 min at room temperature and then for 1 h at 0 °C. The precipitate was collected by filtration, washed with dilute HCl (10 % conc. HCl, 90 % H_2O), and dried under vacuum. The crude product was purified *via* flash column chromatography (20 % - 50 % EtOAc, cyclohexane) to give **24** (334 mg, 13 %) as a light-yellow powder.

mp 105 °C; δ_H (400 MHz, DMSO-d_6) 7.84 - 8.06 (2 H, m, *Ar*), 7.28 - 7.75 (3 H, m, *Ar*), 3.19 (6 H, s, NCH_3); δ_C (100 MHz, DMSO-d_6) 189.8 (*C*=O), 167.8.5 (*C*=O), 150.7, 133.3, 129.7, 129.0, 128.8, 128.1, 96.2 (C_5), 28.2 (N*C*H$_3$); ν_{max}/cm^{-1} 1719 (ketone C=O), 1670 (amide C=O); *m*/*z* (ESI$^-$) 259 ([M-H]$^-$); HRMS (ESI$^-$) $C_{13}H_{11}O_4N_2$, ([M-H]$^-$) calcd 259.0724; found 259.0723.

6-Hydroxy-1,3-dimethyl-5-(2-phenylacetyl)pyrimidine-2,4(1H,3H)-dione (**25**)

Phenylacetyl chloride (1.2 mL, 10 mmol) was slowly added to a stirred solution of 1,3-dimethylbarbituric acid (1.6 g, 10 mmol) in pyridine (6 mL) at room temperature. The resulting reaction mixture was stirred for an additional 2 h at room temperature before being slowly added to a stirred mixture of methanol (6 mL), H_2O (5 mL), and concentrated HCl aq. (15 mL). The resulting suspension was stirred for an additional 30 min at room temperature and then for 1 h at 0 °C. The precipitate was collected by filtration, washed with dilute HCl (10 % conc. HCl, 90 % H_2O), and dried under vacuum. The crude product was purified *via* flash column chromatography (20 % - 50 % EtOAc, cyclohexane) to give **25** (1.67 mg, 61 %) as a light-brown powder.

mp 90 °C; δ_H (400 MHz, DMSO-d_6) 7.23 - 7.38 (5 H, m, *Ar*), 4.49 (2 H, s, CH_2), 3.21 (6 H, s, NCH_3); δ_C (100 MHz, DMSO-d_6) 195.0 (*C*=O), 150.4 (*C*=O), 135.4, 129.9, 128.9, 127.4, 127.0, 96.2 (C_5), 41.3 (CH$_2$), 28.2 (NCH$_3$); ν_{max}/cm^{-1} 1720 (C=O), 1659 (C=O); *m*/*z* (ESI$^-$) 273 ([M-H]$^-$); HRMS (ESI$^-$) $C_{14}H_{14}O_4N_2$, ([M-H]$^-$) calcd 273.0870; found 277.0882.

6-Hydroxy-1,3-dimethyl-5-(3-phenylpropanoyl)pyrimidine-2,4(1H,3H)-dione (**26**)

Hydrocinnamoyl chloride (1.5 mL, 10 mmol) was slowly added to a stirred solution of 1,3-dimethylbarbituric acid (1.6 g, 10 mmol) in pyridine (6 mL) at room temperature. The resulting reaction mixture was stirred for an additional 2 h at room temperature before being slowly added to a stirred mixture of methanol (6 mL), H_2O (5 mL), and concentrated HCl aq. (15 mL). The resulting suspension was stirred for an additional 30 min at room temperature and then for 1 h at 0 °C. The precipitate was collected by filtration, washed with dilute HCl (10 % conc. HCl, 90 % H_2O), and dried under vacuum. The crude product was purified *via* flash column

chromatography (20 % - 50 % EtOAc, cyclohexane) to give **26** (2.07 g, 72 %) as a light-brown powder.

mp 91 °C; δ_H (400 MHz, DMSO-d_6) 7.26 - 7.37 (4 H, m, *Ar*), 7.15 - 7.25 (1 H, m, *Ar*), 3.34 - 3.42 (2 H, m, H_a), 3.21 (6 H, s, NCH_3), 2.87 - 2.98 (2 H, m, H_b); δ_C (100 MHz, DMSO-d_6) 197.1 (*C*=O), 150.4 (*C*=O), 140.9, 128.9, 128.8, 126.7, 95.9 (C_5), 38.4 (C_a), 31.3 (C_b), 28.2 (NCH_3); ν_{max}/cm^{-1} 1719 (ketone C=O), 1664 (amide C=O); *m/z* (ESI^-) 277 ($[M-H]^-$); HRMS (ESI^-) $C_{13}H_{13}O_5N_2$, ($[M-H]^-$) calcd 277.0830; found 277.0838.

General Procedure 1 for the synthesis of substituted barbiturates

The requisite carboxylic acid (1 eq.) was stirred with oxalyl chloride (762 µL, 9 mmol) and DMF (cat., 1 drop) in CH_2Cl_2 (2 mL) for 2 h at room temperature. The solvent and excess oxalyl chloride were subsequently evaporated using a gentle stream of N_2. Then, a solution of 1,3-dimethylbarbituric acid (1 eq.) in pyridine (2 mL) was slowly added to the residue and the resulting reaction mixture was stirred for 2 h at room temperature. The mixture was then slowly transferred into a stirred mixture of methanol (2 mL), H_2O (1.7 mL), and concentrated HCl aq. (5 mL) and stirring was continued for another 1 h at room temperature. If precipitation occurred, the solids were collected by filtration, washed with dilute HCl (10 % conc. HCl, 90 % H_2O), and dried under vacuum. Otherwise, CH_2Cl_2 (15 mL) was added, the mixture was shaken, and the organic layer was collected by passing the mixture through a phase separator cartridge. The organic layer was dried over anhydrous $MgSO_4$ and concentrated in vacuo. The crude product was then purified via flash column chromatography (20 % - 50 % EtOAc, cyclohexane) to give the corresponding triketone product.

6-Hydroxy-1,3-dimethyl-5-(4-phenylbutanoyl)pyrimidine-2,4(1H,3H)-dione (**27**)

Following General Procedure 1, 3-phenylbutyric acid (293 mg, 3 mmol) and 1,3-dimethylbarbituric acid (468 mg, 3 mmol) gave **27** (444 mg, 49 %) as an off-white solid.

mp 68 ^{o}C; δ_H (400 MHz, DMSO-d_6) 7.14 - 7.35 (5 H, m, *Ar*), 3.19 (6 H, s, NCH_3), 3.06 - 3.15 (2 H, m, H_a), 2.68 (2 H, t, *J*=7.5 Hz, H_c), 1.93 (2 H, quin, *J*=7.5 Hz, H_c); δ_C (100 MHz, DMSO-d_6) 197.9 (*C*=O), 150.4 (*C*=O), 141.8, 128.8, 1264., 96.0 (C_5), 35.9 (C_a), 35.2 (C_c), 28.1 (NCH_3), 27.3 (C_b); ν_{max}/cm^{-1} 1716 (ketone C=O), 1648 (amide C=O); *m/z* (ESI$^-$) 301 ([M-H]$^-$); HRMS (ESI$^-$) $C_{16}H_{17}O_4N_2$, ([M-H]$^-$) calcd 301.1194; found 301.1200.

6-Hydroxy-1,3-dimethyl-5-(5-phenylpentanoyl)pyrimidine-2,4(1H,3H)-dione (**28**)

Following General Procedure 1, 5-phenylvaleric acid (534 mg, 3 mmol) and 1,3-dimethylbarbituric acid (468 mg, 3 mmol) gave **28** (519 mg, 55 %) as a light-yellow oil.

δ_H (400 MHz, DMSO-d_6) 7.03 - 7.44 (5 H, m, *Ar*), 3.19 (6 H, s, NCH_3), 3.07 - 3.15 (2 H, m), 2.57 - 2.67 (2 H, m), 1.59 - 1.72 (4 H, m); δ_C (100 MHz, DMSO-d_6) 150.4, 142.3, 128.8, 126.2, 95.9 (C_5), 36.0, 35.2, 31.1, 28.1 (NCH_3), 25.5; ν_{max}/cm^{-1} 1722 (ketone C=O), 1667 (amide C=O); *m/z* (ESI$^-$) 315 ([M-H]$^-$); HRMS (ESI$^-$) $C_{17}H_{19}O_4N_2$, ([M-H]$^-$) calcd 315.1350; found 315.1356.

5-(3-Cyclopentylpropanoyl)-6-hydroxy-1,3-dimethylpyrimidine-2,4(1H,3H)-dione (**29**)

Cyclopentanepropionyl chloride (460 µL, 3 mmol) was slowly added to a solution of 1,3-dimethylbarbituric acid (312 mg, 3 mmol) in pyridine (2 mL) and the resulting reaction mixture was stirred for 2 h at room temperature. The mixture was then slowly transferred into a stirred mixture of methanol (2 mL), H_2O (1.7 mL), and concentrated HCl aq. (5 mL) and stirring was

continued for another 1 h at room temperature. CH_2Cl_2 (15 mL) was added, the mixture was shaken, and the organic layer was collected by passing the mixture through a phase separator cartridge. The organic layer was dried over anhydrous $MgSO_4$ and concentrated *in vacuo*. The crude product was then purified *via* flash column chromatography (20 % - 50 % EtOAc, cyclohexane) to give **29** (361 mg, 42 %) as a light-yellow oil.

δ_H (400 MHz, DMSO-d_6) 3.19 (6 H, s, NCH_3), 3.01 - 3.13 (2 H, m, $COCH_2$), 1.68 - 1.90 (4 H, m), 1.40 - 1.66 (6 H, m); δ_C (100 MHz, DMSO-d_6) 198.7, 150.4, 95.8 (C_5), 35.6, 32.4, 32.2, 28.1 (NCH$_3$), 25.2; ν_{max}/cm^{-1} 1724 (ketone C=O), 1669 (amide C=O); *m/z* (ESI^-) 279 ([M-H]$^-$); HRMS (ESI^-) $C_{14}H_{19}O_4N_2$, ([M-H]$^-$) calcd 279.1350; found 279.1351.

1,3-Diethylpyrimidine-2,4,6(1H,3H,5H)-trione (**S2**)[5]

A stirred solution of malonic acid (5 g, 48 mmol) and 1,3-diethylurea (5 g, 48 mmol) in acetic acid (10 mL) was heated to 60 °C. Acetic anhydride (10 mL) was added to the reaction mixture which was stirred for another 6 h at 60 °C. Then, H_2O is (5 mL) is slowly added, and the mixture was stirred at 70 °C for 30 min. After cooling to room temperature, the reaction volume was reduced and the precipitate was collected by filtration, washed with H_2O, and dried to give **S2** (5.73 g, 65 %) as a white powder.

δ_H (400 MHz, DMSO-d_6) 3.77 (4 H, q, J=7.0 Hz, CH_2), 3.71 (2 H, s, C^5H_2), 1.10 (6 H, t, J=7.0 Hz, CH_3); δ_C (100 MHz, DMSO-d_6) 166.0, 151.9, 36.5 (CH$_2$), 13.4 (CH$_3$); *m/z* (ESI^-) 183 ([M-H]$^-$).

5-(3-Cyclopentylpropanoyl)-1,3-diethyl-6-hydroxypyrimidine-2,4(1H,3H)-dione (30)

Cyclopentanepropionyl chloride (460 μL, 3 mmol) was slowly added to a solution of **S2** (552 mg, 3 mmol) in pyridine (2 mL) and the resulting reaction mixture was stirred for 2 h at room temperature. The mixture was then slowly transferred into a stirred mixture of methanol (2 mL), H_2O (1.7 mL), and concentrated HCl aq. (5 mL) and stirring was continued for another 1 h at room temperature. CH_2Cl_2 (15 mL) was added, the mixture was shaken, and the organic layer was collected by passing the mixture through a phase separator cartridge. The organic layer was dried over anhydrous $MgSO_4$ and concentrated *in vacuo*. The crude product was then purified *via* flash column chromatography (20 % - 50 % EtOAc, cyclohexane) to give **30** (536 mg, 58 %) as a light-yellow oil.

δ_H (400 MHz, DMSO-d_6) 3.76 - 3.93 (4 H, m, NCH_2), 3.01 - 3.20 (2 H, m, H_a), 1.68 - 1.91 (3 H, m), 1.41 - 1.66 (6 H, m), 1.01 - 1.23 (8 H, m); δ_C (100 MHz, DMSO-d_6) 198.7, 149.5, 95.8 (C_5), 35.7, 32.4, 32.1, 25.2, 25.1, 13.4; ν_{max}/cm^{-1} 1720 (ketone C=O), 1667 (amide C=O); *m*/*z* (ESI$^-$) 307 ([M-H]$^-$); HRMS (ESI$^-$) $C_{16}H_{23}O_4N_2$, ([M-H]$^-$) calcd 307.1663; found 307.1667.

1,3-Diisopropylpyrimidine-2,4,6(1H,3H,5H)-trione (S3)[6]

A solution of *N*,*N*'-diisopropylcarbodiimide (3.1 mL, 20 mmol) in THF (10 mL) was slowly added to a solution of malonic acid (1.04 g, 10 mmol) in THF (10 mL) at 0 °C. The resulting reaction mixture was warmed to room temperature and stirred for 2 h. The precipitate was collected by filtration and immediately slurried in ethanol (20 mL) and heated to reflux. The mixture was then allowed to cool to room temperature and the precipitate was filtered, washed with cold ethanol, and dried to give **S3** (1.45 g, 68 %) as a white powder.

δ_H (400 MHz, DMSO-d_6) 5.50 (2 H, d, J=7.5 Hz, C^5H_2), 3.58 - 3.71 (2 H, m, C*H*(CH_3)$_2$), 1.01 (12 H, d, J=6.5 Hz, CH(C*H*$_3$)$_2$; δ_C (100 MHz, DMSO-d_6) 157.3, 41.1 (*C*H(CH_3)$_2$), 23.8 (CH(*C*H$_3$)$_2$); *m/z* (ESI^-) 211 ($[M-H]^-$).

1,3-Dicyclohexylpyrimidine-2,4,6(1H,3H,5H)-trione (**S4**)

A solution of *N*,*N*'-dicyclohexylcarbodiimide (2.54 g, 12.3 mmol) in THF (10 mL) was slowly added to a solution of malonic acid (641 mg, 6.2 mmol) in THF (10 mL) at 0 °C. The resulting reaction mixture was warmed to room temperature and stirred for 2 h. The precipitate was collected by filtration and immediately slurried in ethanol (20 mL) and heated to reflux. The mixture was then allowed to cool to room temperature and the precipitate was filtered, washed with cold ethanol, and dried to give **S4** (1.3 g, 72 %) as a white powder.

mp 203 °C; δ_H (400 MHz, DMSO-d_6) 4.34 - 4.54 (2 H, m, NC*H*), 3.70 (2 H, s, C^5H_2), 2.00 - 2.29 (4 H, m), 1.70 - 1.86 (4 H, m), 1.48 - 1.68 (6 H, m), 1.19 - 1.38 (4 H, m), 1.03 - 1.18 (2 H, m); δ_C (100 MHz, DMSO-d_6) 166.5, 152.0, 54.2, 41.7, 29.1, 26.4, 25.3; ν_{max}/cm^{-1} 1743 (C=O), 1691 (C=O), 1672 (C=O); *m/z* (ESI^-) 291 ($[M-H]^-$); HRMS (ESI^-) $C_{16}H_{23}O_3N_2$, ($[M-H]^-$) calcd 291.1703; found 291.1716.

1,3-Dicyclohexyl-5-(3-cyclopentylpropanoyl)-6-hydroxypyrimidine-2,4(1H,3H)-dione (31)

Cyclopentanepropionyl chloride (460 µL, 3 mmol) was slowly added to a solution of **S4** (876 mg, 3 mmol) in pyridine (6 mL) and the resulting reaction mixture was stirred for 2 h at room temperature. The mixture was then slowly transferred into a stirred mixture of methanol (2 mL), H_2O (1.7 mL), and concentrated HCl aq. (5 mL) and stirring was continued for another 1 h at room temperature. CH_2Cl_2 (15 mL) was added, the mixture was shaken, and the organic

layer was collected by passing the mixture through a phase separator cartridge. The organic layer was dried over anhydrous $MgSO_4$ and concentrated *in vacuo*. The crude product was then purified *via* flash column chromatography (20 % - 50 % EtOAc, cyclohexane) to give **31** (763 mg, 61 %) as a light-yellow oil.

δ_H (400 MHz, DMSO-d_6) 4.45 - 4.73 (2 H, m, NC*H*), 2.97 - 3.13 (2 H, m, COCH_2), 2.12 - 2.37 (5 H, m), 1.44 - 1.92 (22 H, m), 0.98 - 1.36 (4 H, m); δ_C (100 MHz, pyridine-d_5) 199.2 (*C*=O), 96.1 (C_5), 54.4, 40.1, 36.6, 32.4, 32.1, 29.1, 26.5, 25.5, 25.2; ν_{max}/cm^{-1} 1741 (ketone C=O), 1668 (amide C=O); *m*/*z* (ESI$^-$) 415 ([M-H]$^-$); HRMS (ESI$^-$) $C_{24}H_{35}O_4N_2$, ([M-H]$^-$) calcd 415.2602; found 415.2623.

6-Hydroxy-1,3-dimethyl-5-(3-(naphthalen-2-yl)propanoyl)pyrimidine-2,4(1H,3H)-dione (32)

Following General Procedure 1, 3-(naphthalene-2-yl)propanoic acid (244 mg, 1.2 mmol) and 1,3-dimethylbarbituric acid (190 mg, 1.2 mmol) gave **32** (176 mg, 43 %) as an off-white powder.

mp 157 °C; δ_H (400 MHz, pyridine-d_5) 9.32 - 9.40 (3 H, m, *Ar*), 9.08 - 9.12 (1 H, m, *Ar*), 8.87 - 9.01 (3 H, m, *Ar*), 5.13 - 5.23 (2 H, m, H_a), 4.84 (6 H, s, NCH_3), 4.71 - 4.81 (2 H, m, H_b); δ_C (100 MHz, DMSO-d_6) 197.3 (*C*=O), 134.0, 132.5, 128.3, 127.9, 127.8, 127.6, 126.9, 126.3, 125.6, 122.9, 95.9 (C_5), 39.3 (C_a), 31.9 (C_b), 27.6 (N*C*H_3); ν_{max}/cm^{-1} 1721 (C=O), 1699 (C=O); *m*/*z* (ESI$^-$) 337 ([M-H]$^-$); HRMS (ESI$^-$) $C_{19}H_{17}O_4N_2$, ([M-H]$^-$) calcd 337.1194; found 337.1203.

6-Hydroxy-1,3-dimethyl-5-(3-(naphthalen-1-yl)propanoyl)pyrimidine-2,4(1H,3H)-dione (33)

Following General Procedure 1, 3-(1-naphthyl)propanoic acid (600 mg, 3 mmol) and 1,3-dimethylbarbituric acid (468 mg, 3 mmol) gave **33** (506 mg, 50 %) as a yellow powder.

mp 179 °C; δ_H (400 MHz, pyridine-d_5) 9.39 - 9.48 (1 H, m, *Ar*), 9.25 - 9.34 (1 H, m, *Ar*), 8.97 - 9.05 (3 H, m, *Ar*), 8.88 - 8.96 (2 H, m, *Ar*), 5.17 - 5.25 (2 H, m, H_a), 5.04 - 5.16 (2 H, m, H_b), 4.84 (6 H, s, NCH_3); δ_C (100 MHz, DMSO-d_6) 198.4 (*C*=O), 138.5, 136.9, 136.0, 135.4, 133.3, 130.2, 128.5, 127.7, 127.6, 127.1, 97.1 (C_5), 39.8 (C_a), 30.1 (C_b), 28.8 (NCH_3); ν_{max}/cm^{-1} 1717 (C=O), 1699 (C=O); *m*/*z* (ESI^-) 337 ($[M-H]^-$); HRMS (ESI^-) $C_{19}H_{17}O_4N_2$, ($[M-H]^-$) calcd 337.1194; found 337.1204.

5-(3-(Furan-3-yl)propanoyl)-6-hydroxy-1,3-dimethylpyrimidine-2,4(1H,3H)-dione (34)

Following General Procedure 1, 3-(furan-3-yl)propanoic acid (240 mg, 1.7 mmol) and 1,3-dimethylbarbituric acid (267 mg, 1.7 mmol) gave **34** (11 mg, 2 %) as a light-pink oil.

δ_H (400 MHz, DMSO-d_6) 7.56 - 7.61 (1 H, m, *Ar*), 7.46 - 7.53 (1 H, m, *Ar*), 6.41 - 6.47 (1 H, m, *Ar*), 3.31 - 3.41 (2 H, m, H_a), 3.21 (6 H, s, NCH_3), 2.67 - 2.81 (2 H, m, H_b); δ_C (100 MHz, DMSO-d_6) 150.4, 143.7, 139.7, 128.9, 111.6, 96.0, 36.9, 28.2(NCH_3); ν_{max}/cm^{-1} 3422 (OH), 1653 (C=O); *m*/*z* (ESI^-) 277 ($[M-H]^-$); HRMS (ESI^-) $C_{13}H_{13}N_2O_5$, ($[M-H]^-$) calcd 277.0830; found 277.0837.

6-Hydroxy-1,3-dimethyl-5-(3-(o-tolyl)propanoyl)pyrimidine-2,4(1H,3H)-dione (35)

Following General Procedure 1, 2-methylhydrocinnamic acid (492 mg, 3 mmol) and 1,3-dimethylbarbituric acid (468 mg, 3 mmol) gave **35** (466 mg, 51 %) as a white solid.

mp 125 °C; δ_H (400 MHz, DMSO-d_6) 7.05 - 7.28 (4 H, m, *Ar*), 3.28 - 3.39 (2 H, m, H_a), 3.21 (6 H, s, NCH_3) 2.80 - 3.00 (2 H, m, H_a), 2.32 (3 H, s, CH_3); δ_C (100 MHz, DMSO-d_6) 197.0 (*C*=O), 150.4, 138.9, 136.2, 130.5, 129.0, 126.8, 126.5, 96.0 (C_5), 37.0 (C_a), 28.9 (C_b), 28.2 (NCH_3), 19.3 (CH_3); ν_{max}/cm^{-1} 1717 (ketone C=O), 1662 (amide C=O); *m*/*z* (ESI^-) 301 ([M-H]$^-$); HRMS (ESI^-) $C_{16}H_{17}O_4N_2$, ([M-H]$^-$) calcd 301.1194; found 301.1192.

6-Hydroxy-1,3-dimethyl-5-(3-(m-tolyl)propanoyl)pyrimidine-2,4(1H,3H)-dione (**36**)

Following General Procedure 1, 3-methylhydrocinnamic acid (492 mg, 3 mmol) and 1,3-dimethylbarbituric acid (468 mg, 3 mmol) gave **36** (506 mg, 56 %) as a white powder.

mp 131 °C; δ_H (400 MHz, pyridine-d_5) 7.15 - 7.25 (4 H, m, *Ar*), 3.52 - 3.61 (2 H, m, H_a), 3.31 (6 H, s, NCH_3), 2.97 - 3.13 (2 H, m, H_b), 2.21 (3 H, s, CH_3); δ_C (100 MHz, pyridine-d_5) 197.4 (*C*=O), 150.3 (*C*=O), 141.1, 138.0, 129.5, 128.6, 127.1, 125.8, 95.8 (C_5), 39.3 (C_a), 31.6 (C_b), 27.6 (NCH_3), 21.1 (CH_3); ν_{max}/cm^{-1} 1717 (C=O), 1661 (C=O); *m*/*z* (ESI^-) 301 ([M-H]$^-$); HRMS (ESI^-) $C_{16}H_{17}O_4N_2$, ([M-H]$^-$) calcd 301.1194; found 301.1200.

6-Hydroxy-1,3-dimethyl-5-(3-(p-tolyl)propanoyl)pyrimidine-2,4(1H,3H)-dione (**37**)

Following General Procedure 1, 4-methylhydrocinnamic acid (492 mg, 3 mmol) and 1,3-dimethylbarbituric acid (468 mg, 3 mmol) gave **37** (565 mg, 62 %) as a white solid.

mp 140 °C; δ_H (400 MHz, DMSO-d_6) 7.03 - 7.23 (4 H, m, *Ar*), 3.32 - 3.39 (2 H, m, H_a), 3.21 (6 H, s, NCH_3), 2.84 - 2.93 (2 H, m, H_b), 2.28 (3 H, s, CH_3); δ_C (100 MHz, DMSO-d_6) 150.4, 137.8, 135.6, 129.4, 129.3, 128.6, 128.5, 96.0 (C_5), 38.4 (C_a), 31.0 (C_b), 28.2 (NCH_3), 21.1 (CH_3); ν_{max}/cm^{-1} 1720 (ketone C=O), 1699 (amide C=O); *m*/*z* (ESI^-) 301 ([M-H]$^-$); HRMS (ESI^-) $C_{16}H_{17}O_4N_2$, ([M-H]$^-$) calcd 301.1194; found 301.1200.

5-(3-Cyclohexylpropanoyl)-6-hydroxy-1,3-dimethylpyrimidine-2,4(1H,3H)-dione (**38**)

Following General Procedure 1, cyclohexanepropionic acid (468 μL, 3 mmol) and 1,3-dimethylbarbituric acid (468 mg, 3 mmol) gave **38** (444 mg, 61 %) as a white solid.

mp 58 °C; δ_H (400 MHz, DMSO-d_6) (6 H, s, NCH_3), 3.02 - 3.12 (2 H, m, H_a), 1.57 - 1.79 (6 H, m), 1.45 - 1.55 (2 H, m, H_b), 1.06 - 1.26 (3 H, m), 0.91 (2 H, m); δ_C (100 MHz, DMSO-d_6) 198.6, 150.4, 95.8 (C_5), 37.5, 33.9, 33.3, 33.0, 28.1, 26.5, 26.2 (NCH$_3$); ν_{max}/cm^{-1} 1720 (ketone C=O), 1670 (amide C=O); *m/z* (ESI$^-$) 293 ([M-H]$^-$); HRMS (ESI$^-$) $C_{15}H_{21}O_4N_2$, ([M-H]$^-$) calcd 293.1507; found 293.1511.

5-(3-Cyclobutylpropanoyl)-6-hydroxy-1,3-dimethylpyrimidine-2,4(1H,3H)-dione (**39**)

Following General Procedure 1, 3-cyclobutylpropanoic acid (250 mg, 2 mmol) and 1,3-dimethylbarbituric acid (312 mg, 2 mmol) gave **39** (135 mg, 17 %) as a light-yellow oil.

δ_H (400 MHz, DMSO-d_6) 3.20 (6 H, s, NCH_3), 2.87 - 3.06 (2 H, m, H_a), 2.32 (1 H, quin, J=8.0 Hz, H_c), 1.95 - 2.09 (2 H, m, H_b), 1.75 - 1.91 (2 H, m, H_e), 1.53 - 1.74 (4 H, m, H_d); δ_C (100 MHz, DMSO-d_6) 198.2, 150.4, 95.8 (C_5), 35.6, 34.2, 32.9, 28.1, 27.9, 18.2; ν_{max}/cm^{-1} 1723 (ketone C=O), 1668 (amide C=O); *m/z* (ESI$^-$) 265 ([M-H]$^-$); HRMS (ESI$^-$) $C_{13}H_{17}O_4N_2$, ([M-H]$^-$) calcd 265.1194; found 265.1192.

6-Hydroxy-1,3-dimethyl-5-(3-(1,2,3,4-tetrahydronaphthalen-2-yl)propanoyl)pyrimidine-2,4(1H,3H)-dione (**40**)

Following General Procedure 1, 1,2,3,4-tetrahydro-2-naphthoic acid (528 mg, 3 mmol) and 1,3-dimethylbarbituric acid (468 mg, 3 mmol) gave **40** (485 mg, 47 %) as an off-white solid.

mp 121 °C; δ_H (400 MHz, DMSO-d_6) 6.96 - 7.19 (4 H, m, *Ar*), 3.21 (6 H, s, NCH_3), 2.90 - 2.99 (2 H, m, H_a), 2.72 - 2.89 (4 H, m), 2.01 - 2.17 (2 H, m, H_b), 1.65 - 1.90 (2 H, m); δ_C (100 MHz, DMSO-d_6) 200.5, 176.7, 150.3, 135.9, 135.3, 129.3, 129.2, 126.3, 126.1, 95.3 (C_5), 31.7, 31.6, 28.8, 28.2 (NCH_3), 26.8, 26.4, 25.9; ν_{max}/cm^{-1} 1721 (ketone C=O), 1655 (amide C=O); A molecular ion for **37** was not observed by either ESI or FI MS analysis.

6-Hydroxy-1,3-dimethyl-5-((1*S*,2*R*)-2-phenylcyclopropane-1-carbonyl)pyrimidine-2,4(1*H*,3*H*)-dione (41)

Following General Procedure 1, *trans*-2-phenylcyclopropane-1-carboxylic acid (486 mg, 3 mmol) and 1,3-dimethylbarbituric acid (468 mg, 3 mmol) gave **41** (419 mg, 47 %) as a white solid.

mp 111 °C; δ_H (400 MHz, DMSO-d_6) 7.07 - 7.44 (5 H, m, *Ar*), 3.18 (6 H, s, NCH_3), 2.75 - 2.96 (1 H, m, H_a), 1.91 - 2.05 (1 H, m, H_b), 1.75 - 1.89 (2 H, m, H_c); δ_C (100 MHz, DMSO-d_6) 150.3, 140.2, 129.0, 128.8, 127.2, 126.6, 126.4, 95.9 (C_5), 31.8, 28.2, 27.3, 20.7; ν_{max}/cm^{-1} 1716 (ketone C=O), 1672 (amide C=O); *m/z* (ESI$^-$) 299 ([M-H]$^-$); HRMS (ESI$^-$) $C_{16}H_{15}O_4N_2$, ([M-H]$^-$) calcd 299.1037; found 299.1041.

6-Hydroxy-1,3-dimethyl-5-(3-(thiophen-2-yl)propanoyl)pyrimidine-2,4(1H,3H)-dione (42)

Following General Procedure 1, 2-thiophenepropionic acid (468 mg, 3 mmol) and 1,3-dimethylbarbituric acid (468 mg, 3 mmol) gave **42** (371 mg, 42 %) as a light-yellow solid.

mp 91 °C; δ_H (400 MHz, DMSO-d_6) 7.23 - 7.42 (1 H, m, *Ar*), 6.84 - 7.05 (2 H, m, *Ar*), 3.37 - 3.56 (2 H, m, H_a), 3.21 (6 H, s, NCH_3), 3.14 - 3.19 (2 H, m, H_b); δ_C (100 MHz, DMSO-d_6) 196.4, 150.4, 143.2, 127.4, 125.5, 124.6, 96.0 (C_5), 38.5 (C_a), 28.2 (NCH_3), 25.3 (C_b); ν_{max}/cm$^-$

[1] 1716 (ketone C=O), 1661 (amide C=O); *m*/*z* (ESI^-) 293 ($[M-H]^-$); HRMS (ESI^-) $C_{13}H_{13}O_4N_2S$, ($[M-H]^-$) calcd 293.0602; found 293.0602.

5-(2,3-Dihydro-1H-indene-2-carbonyl)-6-hydroxy-1,3-dimethylpyrimidine-2,4(1H,3H)-dione (43)

Following General Procedure 1, indan-2-carboxylic acid (486 mg, 3 mmol) and 1,3-dimethylbarbituric acid (468 mg, 3 mmol) gave **43** (454 mg, 50 %) as an off-white solid.

mp 120 °C; δ_H (400 MHz, DMSO-d_6) 7.02 - 7.44 (4 H, m, *Ar*), 4.69 - 4.99 (1 H, m, H_a), 3.22 (6 H, s, NCH_3), 3.10 - 3.33 (4 H, m, H_b); δ_C (100 MHz, DMSO-d_6) 199.6, 150.3, 141.8, 127.0, 124.7, 95.3 (C_5), 43.8 (C_a), 36.4 (C_b), 28.2 (N*C*H_3); ν_{max}/cm^{-1} 1718 (ketone C=O), 1663 (amide C=O); *m*/*z* (ESI^-) 299 ($[M-H]^-$); HRMS (ESI^-) $C_{16}H_{15}O_4N_2$, ($[M-H]^-$) calcd 299.1037; found 299.1043.

6-Hydroxy-1,3-dimethyl-5-(3-phenylbutanoyl)pyrimidine-2,4(1H,3H)-dione (44)

Following General Procedure 1, 3-phenylbutyric acid (492 mg, 3 mmol) and 1,3-dimethylbarbituric acid (468 mg, 3 mmol) gave **44** (316 mg, 35 %) as an off-white wax.

mp 100 °C; δ_H (400 MHz, DMSO-d_6) 7.24 - 7.33 (5 H, m, *Ar*), 3.39 - 3.47 (1 H, m, C*H*), 3.26 - 3.37 (2 H, m, CH_2), 3.20 (6 H, s, NCH_3), 1.25 - 1.33 (3 H, m, CH_3); δ_C (100 MHz, DMSO-d_6) 196.7, 150.4, 146.1, 128.8, 127.2, 126.8, 96.5 (C_5), 44.3 (*C*H), 37.1 (*C*H_2), 28.2 (N*C*H_3), 22.1 (*C*H_3); ν_{max}/cm^{-1} 1706 (ketone C=O), 1655 (amide C=O); *m*/*z* (ESI^-) 301 ($[M-H]^-$); HRMS (ESI^-) $C_{16}H_{17}O_4N_2$, ($[M-H]^-$) calcd 301.1194; found 301.1200.

6-Hydroxy-1,3-dimethyl-5-(2-methyl-3-phenylpropanoyl)pyrimidine-2,4(1H,3H)-dione (45)

Following General Procedure 1, α-methylhydrocinnamic acid (492 mg, 3 mmol) and 1,3-dimethylbarbituric acid (468 mg, 3 mmol) gave **45** (378 mg, 42 %) as an off-white wax.

δ_H (400 MHz, DMSO-d_6) 7.04 - 7.42 (5 H, m, *Ar*), 3.21 (6 H, s, NCH_3), 2.95 - 3.11 (1 H, m, C*H*), 2.54 - 2.77 (2 H, m, CH_2), 1.10 (3 H, d, *J*=7.0 Hz, CH_3); δ_C (100 MHz, DMSO-d_6) 177.3, 150.3, 140.1, 139.7, 129.4, 128.7, 126.5, 95.3 (C_5), 41.1 (*C*H), 28.2 (N*C*H_3), 17.1, 17.0; ν_{max}/cm^{-1} 1720 (ketone C=O), 1654 (amide C=O); *m/z* (ESI$^-$) 301 ([M-H]$^-$); HRMS (ESI$^-$) $C_{16}H_{17}O_4N_2$, ([M-H]$^-$) calcd 301.1194; found 301.1197.

N-Benzyl-6-hydroxy-1,3-dimethyl-2,4-dioxo-1,2,3,4-tetrahydropyrimidine-5-carboxamide (46)

A solution of benzyl isocyanate (605 µL, 5 mmol) in CH_2Cl_2 (5 mL) was added dropwise to a stirred solution of 1,3-dimethylbarbituric acid (780 mg, 5 mmol) and diisopropylethylamine (1.74 mL, 10 mmol) in CH_2Cl_2 (15 mL). The resulting reaction mixture was stirred for 16 h at room temperature before HCl (6 N aq., 5 mL) was added dropwise. The organic layer was separated, dried over anhydrous $MgSO_4$, and concentrated *in vacuo*. The crude product was purified *via* flash column chromatography (20 % - 50 % EtOAc, cyclohexane) to give **46** (1.09 g, 75 %) as an off-white powder.

mp 103 °C; δ_H (400 MHz, DMSO-d_6) 7.08 - 7.63 (5 H, m, *Ar*), 4.60 (2 H, d, *J*=6.0 Hz, CH_2), 3.18 (6 H, s, NCH_3); δ_C (100 MHz, DMSO-d_6) 170.6, 150.4, 137.9, 129.1, 127.9, 79.8 (C_5), 43.5 (*C*H_2), 27.8 (N*C*H_3); ν_{max}/cm^{-1} 1697 (ketone C=O), 1638 (amide C=O); *m/z* (ESI$^-$) 288 ([M-H]$^-$); HRMS (ESI$^-$) $C_{14}H_{10}O_4N_3$, ([M-H]$^-$) calcd 288.0990; found 288.0994.

Enzyme Inhibition Assays

OGFOD1 inhibition assays were performed by determining the extent of hydroxylation of a 20mer fragment of human ribosomal protein S23 (RPS23) containing residues 51-70 (H_2N-VLEKVGVEAKQPNSAIRKCV-$CONH_2$) by matrix-assisted laser desorption/ionization time-of-flight mass spectrometry (MALDI-TOF MS) using a Waters Micromass MALDI micro MX mass spectrometer and MassLynx 4.1, as previously described.[7,8] The optimized hydroxylation assay involved incubation of OGFOD1 (1 μM) with inhibitor (1 % *v*/*v* in DMSO) in the presence of $(NH_4)_2Fe(SO_4)_2$ (50 μM), the disodium salt of 2-oxoglutarate (25 μM), sodium ascorbate (100 μM), and $RPS23_{51\text{-}70}$ (25 μM) in HEPES (50 mM, pH 7.5) at 37 °C. OGFOD1 was pre-incubated with inhibitor for 5 min before initiation of the reaction via the addition of the other reagents. Reactions were quenched with formic acid (1 % *v*/*v*) after 15 min. Samples were prepared for MALDI using α-cyano-4-hydrocinnamic acid (CHCA; prepared in 50 % acetonitrile) as a matrix. Dose-responses were assessed in 8-point triplicates, normalized to a no enzyme negative control and a no inhibitor positive control. Non-linear regression analysis was performed using Prism (GraphPad).

$PHD2_{181\text{-}426}$ inhibition assays were performed by determining the extent of hydroxylation of a HIF-1α CODD peptide substrate (HIF-1α residues 556-574), using MALDI-TOF MS as described with OGFOD1.[8] The optimized hydroxylation assay involved incubation of $PHD2_{181\text{-}426}$ (1 μM) with inhibitor (1 % *v*/*v* in DMSO) in the presence of $(NH_4)_2Fe(SO_4)_2$ (10 μM), the disodium salt of 2-oxoglutarate (60 μM), sodium ascorbate (100 μM), and HIF-1α$CODD_{556\text{-}574}$ (50 μM) in HEPES (50 mM, pH 7.5) at 37 °C for 15 min. The FIH assay employed MALDI-TOF MS detection of hydroxylation. Reactions consisted of FIH (50 nM), ankyrin peptide (50 μM; HLEVVKLLLEAGADVNAQDK), the disodium salt of 2-oxoglutarate (100 μM), sodium ascorbate (100 μM), and $(NH_4)_2Fe(SO_4)_2$ (10 μM), and

reactions were quenched after 4 min. KDM4A inhibition was tested as described previously using a formaldehyde dehydrogenase (FDH) assay.[9] JARID1B and JARID1C inhibition was tested as described previously using reported AlphaScreen-based assays.[10]

MINA53$_{1-464}$ and NO66$_{116-641}$ were cloned into pFB-LIC-Bse, and the N-terminal His-tagged proteins were produced in *Sf9* insect cells. The MINA53 peptide substrate Rpl27a (Gly31 – Pro49 GRGNAGGLHHHRINFDKYHP) and the NO66 peptide substrate Rpl8 (Asn205 – Thr224 NPVEHPFGGGNHQHIGKPST) were synthesized by Peptide Protein Research Ltd (Fareham, Hampshire, UK) to >95% purity. IC_{50} determinations were performed in 384-well polypropylene plates (Greiner Bio One, Cat Number 781096), and compound dispenses were performed using an ECHO 550 acoustic dispenser (Labcyte, Sunnyvale, CA). NO66 (diluted to 375 nM in 50 mM MES pH 7.0, 150 mM NaCl) and MINA53 (diluted to 187.5 nM in 50 mM HEPES pH 7.5, 50 mM NaCl) were dispensed (40 µL) into 384-well compound plates using a multidrop combi reagent dispenser (Thermo Scientific, Code 5840300) with a small tube plastic tip dispensing cassette (Thermo Scientific, Code 24073290). NO66 and MINA53 were pre-incubated with compound for 15 minutes, and the enzyme reaction initiated by dispensing 10 µL of substrate solution [250 µM $(NH_4)_2Fe(SO_4)_2$, 500 µM sodium ascorbate, 25 µM 2OG, 25 µM peptide] in optimized enzyme buffer. The enzyme reactions were quenched by addition of 10 % formic acid (5 µL) after 30 min (MINA53) or 60 min (NO66), and the assay plates were transferred to a RapidFire RF 360. Samples were aspirated under vacuum, loaded onto a C4 solid phase extraction (SPE) cartridge, and washed for 5.5 s with 0.1% (v/v) formic acid in LCMS grade water to remove non-volatile buffer components. Analytes were then eluted onto an Agilent 6530 accurate mass Q-TOF using 85% acetonitrile in LCMS grade water containing 0.1% formic acid. Ion data for the substrate and hydroxylated (+16) peptide product was extracted, and peak area data were integrated using

RapidFire integrator software (Agilent). Percent conversion was calculated in Excel, and IC_{50} curves were generated using Prism v. 7.0 (GraphPad).

Differential Scanning Fluorimetry (DSF) Assays

Assays consisted of enzyme (2 μM), inhibitor (200 μM), $MnCl_2$ (50 μM), and SYPRO Orange (1:5000 dilution), all prepared in 50 mM HEPES, pH 7.5. Assays were conducted using white 96-well RT-PCR plates. An Opticon thermal cycler (MJ Research Inc) was used to control temperature and measure fluorescence. Data were processed using the OpticonMONITOR Analysis Software, while curve fitting was performed in Prism (GraphPad).

Modelling

Stable small-molecule inhibitor conformations were investigated using the MM2 force field method in ChemBio3D 11.0 (PerkinElmer Informatics). Protein structures were aligned using PyMOL version 2.0 (Schrödinger, LLC). The small-molecule inhibitors were then modelled into the protein active sites, overlaying them with the already contained inhibitor, if applicable (e.g., FG2216 with PHD2, PDB 3HQU; *N*-oxalylglycine with OGFOD1, PDB 4NHX). Care was taken to ensure bidentate metal chelation, steric constraints, and the salt bridge interaction with the active site arginine, if applicable.

NMR spectroscopy

NMR studies were carried out using a Bruker AVIII 700 MHz spectrometer equipped with a 5 mm $^{1}H(^{13}C/^{15}N)$ inverse cryoprobe. Data were processed with Bruker 3.1 software.

CPMG Binding Experiments: Binding of the inhibitors to the proteins was monitored using Carr-Purcell-Meiboom-Gill (CPMG)-edited ^{1}H spectra. The PROJECT-CPMG sequence was applied as described by Aguilar *et al.*[11] The standard experimental parameters used were a

total echo time of 40 ms, a relaxation delay of 2 s, with 256 scans. Water suppression was achieved by presaturation. Samples contained 30 μM *apo*-$PHD2_{181\text{-}426}$ or OGFOD1, supplemented with 60 μM Zn(II), 30 μM of inhibitor, all in 50 mM Tris-d_{11}, pH 7.5, 10 % D_2O, 90 % H_2O with 0.02 % NaN_3.

wLOGSY Binding Experiments: For water-ligand observed gradient spectroscopy (wLOGSY) analyses, typical experimental parameters were the following: mixing time, 1 s; relaxation delay, 2 s; number of transients, 512. Solvent excitation was achieved using a 16-ms 180° selective rectangular shape pulse with 1,000 points (Squa100.1000) set at the H_2O frequency. Water suppression was achieved by a 2-ms sinc pulse (Sinc1.1000) at the H_2O frequency. Samples contained 5-fold molar excess inhibitor:protein as detailed in the figure legends; all analyses were in 50 mM Tris-d_{11}, pH 7.5, 10 % D_2O, 90 % H_2O supplemented with 60 μM Zn(II) and 0.02 % NaN_3.

Competition Experiments by 2OG Displacement: 2OG displacement assays[12] were performed using CPMG-edited ^{1}H spectra. The PROJECT-CPMG sequence was applied as described by Aguilar *et al.*[11] The standard experimental parameters used were a total echo time of 40 ms, a relaxation delay of 2 s, with 160 scans. Water suppression was achieved by presaturation. The 2OG methylene resonance at 2.35 ppm was monitored. The percentage 2OG displacement was defined as:

$$\%\ 2\text{OG displacement} = \frac{I_{2\text{OG}} - I_{2\text{OG}(0)}}{I_{2\text{OG(blank)}} - I_{2\text{OG}(0)}} \times 100$$

where I_{2OG} is the integral of the 2OG ^{1}H signal in the presence of both the inhibitor and the protein, $I_{2OG(0)}$ is the integral of 2OG in the presence of the protein but in the absence of the inhibitor, and $I_{2OG(blank)}$ is the integral of 2OG in the absence of both protein and inhibitor. Samples contained 10 μM 2-oxoglutarate disodium salt, 10 μM *apo*-$PHD2_{181\text{-}426}$ or OGFOD1,

supplemented with 80 μM Zn(II), 70 μM of inhibitor, all in 50 mM Tris-d_{11}, pH 7.5, 10 % D_2O, 90 % H_2O with 0.02 % NaN_3.

Cell Culture and Immunoblotting

HeLa cells were cultured in DMEM media supplemented with 10 % fetal bovine serum, 4 mM L-glutamine, 100 U/mL penicillin, and 100 μg/mL streptomycin. Cells were treated with the indicated compounds, prepared in 1% DMSO, for 5 h. Following a PBS buffer rinse, cells were lysed in urea/SDS buffer supplemented with 1 mM dithiothreitol. Cell extracts were analyzed by SDS-PAGE, electroblotted onto PVDF membranes (Millipore) and probed with anti-HIF-1α antibody (clone 54, BD Transduction Laboratories) at 1:1000 dilution for 1 h at room temperature. The HRP-conjugated secondary antibody was then applied at room temperature for 1 h. Target proteins were detected with SuperSignal Chemiluminescent Substrates (Pierce).

ADMET in vitro Predictors

S9 liver microsome stability assays and MDCK-MDR1 efflux assays were carried out for **CCT3** and **CCT4** by Cyprotex (UK). Additional details are provided later in the Supporting Information.

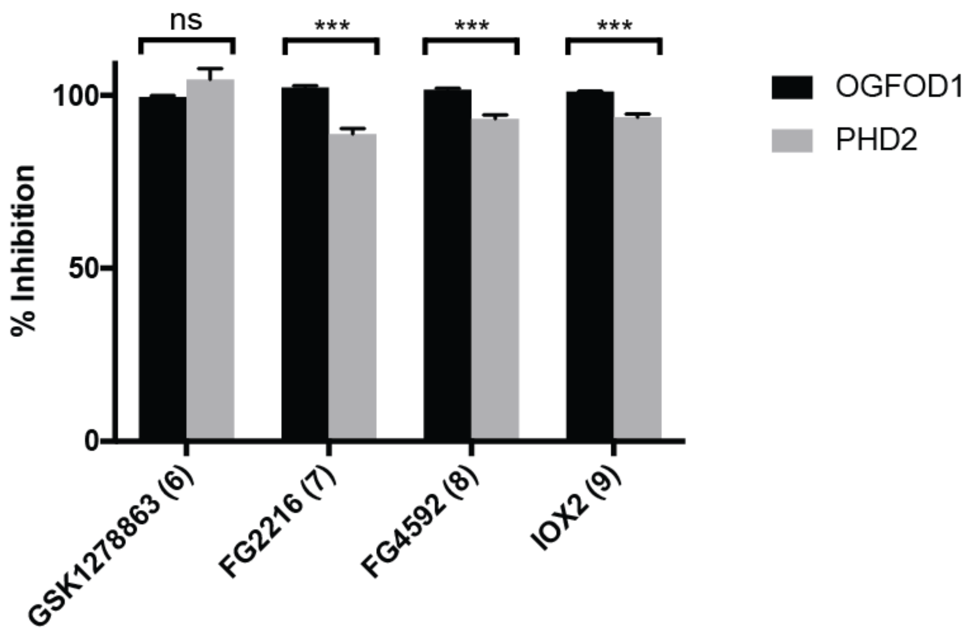

Figure S1. Impact of PHD inhibitors on OGFOD1 and PHD2 activity. The impact of GSK1278863 (**6**), FG2216 (**7**), FG4592 (**8**), and IOX2 (**9**) (100 µM) on the hydroxylation activity of 2-oxoglutarate and iron-dependent oxygenase domain containing 1 (OGFOD1; 1 µM) and prolyl hydroxylase domain-containing protein 2 ($PHD2_{181-426}$; 1 µM) was tested under standard assay conditions (see Experimental section). The plotted data represent the mean percentage inhibition for the experiment performed in triplicate, while the error bars indicate the standard deviation. Note that IC_{50} values for these inhibitors against OGFOD1 and PHD2 have been recently reported by us.[13] Statistical significance was examined with an unpaired t-test in Prism 7 (GraphPad). *** = P-value ≤ 0.001, ns = P-value > 0.05.

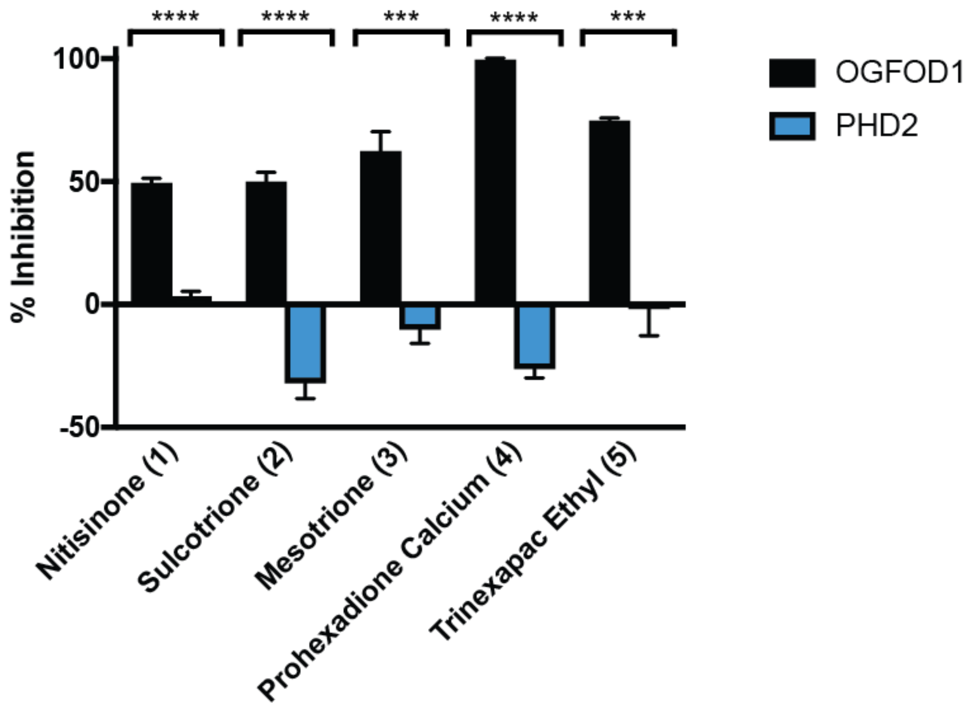

Figure S2. Impact of HPPD (and plant growth) inhibitors on OGFOD1 and PHD2 activity. The impact of nitisinone (**1**), sulcotrione (**2**), mesotrione (**3**), prohexadione calcium (**4**), and trinexapac ethyl (**5**) (100 µM) on the hydroxylation activity of OGFOD1 (1 µM) and $PHD2_{181-426}$ (1 µM) was tested under standard assay conditions (see Experimental section). Note that the negative percent inhibition values observed for PHD2 may indicate that these ligands increase the enzymatic activity of PHD2/stabilise active PHD2 under these assay conditions.[14] The plotted data represent the mean percentage inhibition for the experiment performed in triplicate; the error bars indicate the standard deviation. Statistical significance was examined with an unpaired t-test in Prism 7 (GraphPad). **** = P-value ≤ 0.0001, *** = P-value ≤ 0.001.

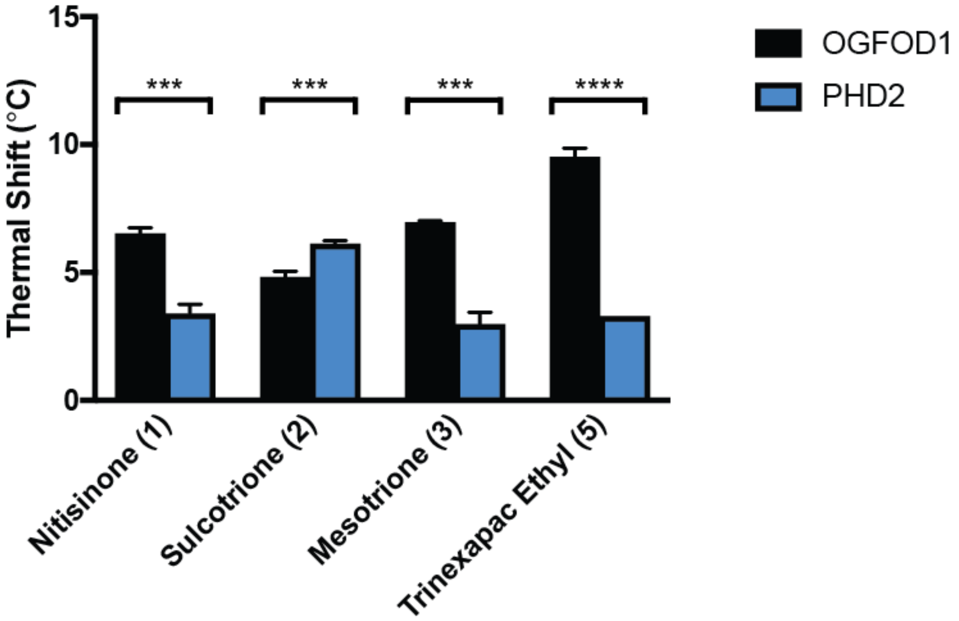

Figure S3. Apparent thermal stabilization of OGFOD1 and PHD2 by HPPD (and plant growth) inhibitors. Thermal stabilization of OGFOD1 (2 μM) and PHD2$_{181\text{-}426}$ (2 μM) in the presence of 200 μM nitisinone (**1**), sulcotrione (**2**), mesotrione (**3**), and trinexapac ethyl (**5**), as determined by differential scanning fluorimetry (see Experimental section for further details). The plotted data represent the mean percentage thermal shift for the experiment performed in triplicate; the error bars indicate the standard deviation. Statistical significance was examined with an unpaired t-test in Prism 7 (GraphPad). **** = P-value ≤ 0.0001, *** = P-value ≤ 0.001.

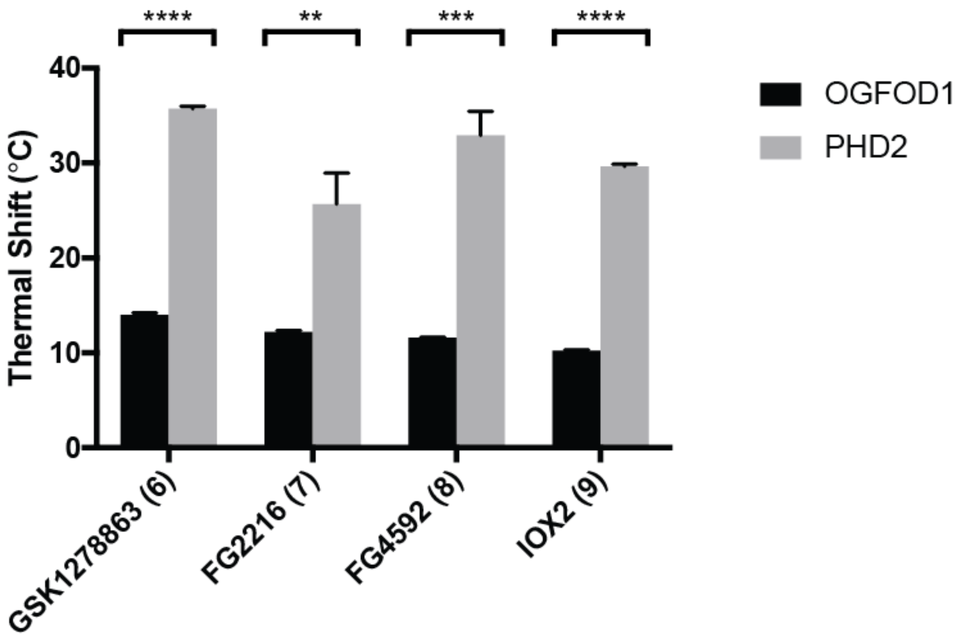

Figure S4. Apparent thermal stabilization of OGFOD1 and PHD2 by glycinamide-containing inhibitors. Thermal stabilization of OGFOD1 (2 μM) and $PHD2_{181\text{-}426}$ (2 μM) in the presence of 200 μM GSK1278863 (**6**), FG2216 (**7**), FG4592 (**8**), and IOX2 (**9**), as determined by differential scanning fluorimetry (see Experimental section for further details). The plotted data represent the mean percentage thermal shift for the experiment performed in triplicate; the error bars indicate the standard deviation. Statistical significance was examined with an unpaired t-test in Prism 7 (GraphPad). **** = P-value ≤ 0.0001, *** = P-value ≤ 0.001, ** = P-value ≤ 0.01.

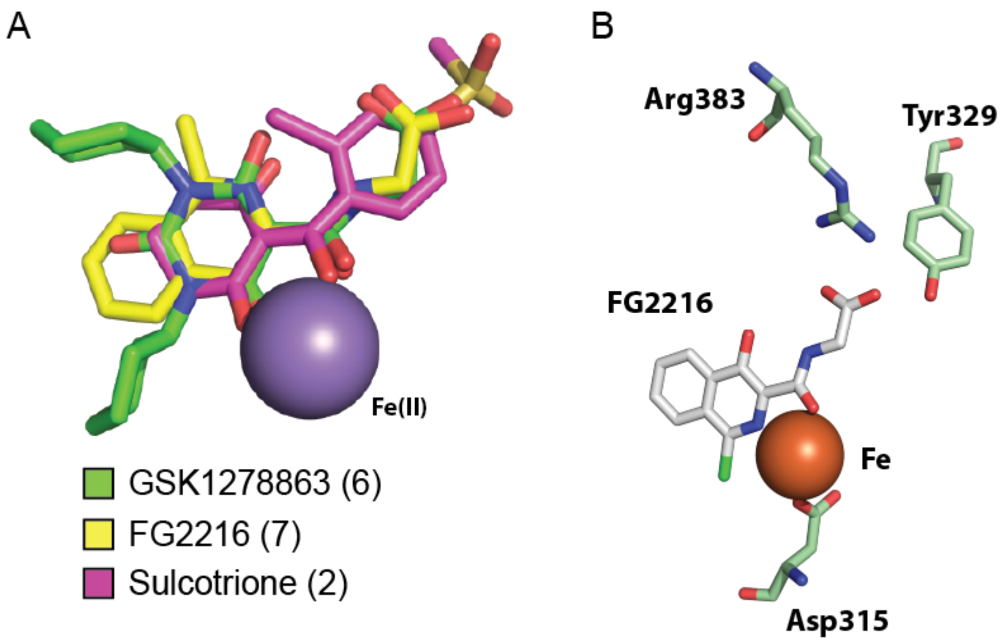

Figure S5. (A) Comparison of the chelation motifs of sulcotrione **2** and GSK1278863 **6**, modelled based on the crystallographically observed structure of PHD2 with bound FG2216 **7** (PDB 3HQU).[15] (B) View from the active site of the PHD2:FG2216 **7** complex, showing the interaction of the iron with Asp315, and the interaction of the FG2216 glycinamide side chain with Tyr329 and Arg383 (PDB 3HQU).[15]

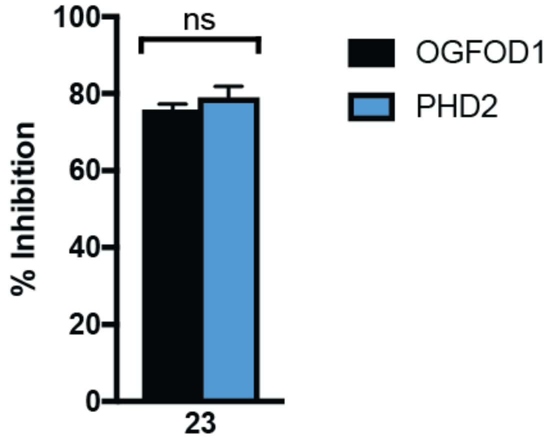

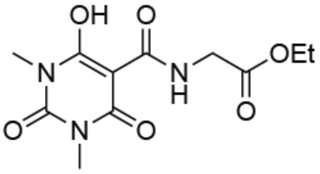

23

Figure S6. Inhibition of PHD2 and OGFOD1 by 23. The selectivity of the inhibition of OGFOD1 (1 μM) and $PHD2_{181-426}$ (1 μM) by **23** (100 μM) was tested under standard assay conditions (see Experimental section). The plotted data represent the mean percentage inhibition for the experiment performed in triplicate; the error bars indicate the standard deviation. Statistical significance was examined with an unpaired t-test in Prism 7 (GraphPad). ns = P-value > 0.05.

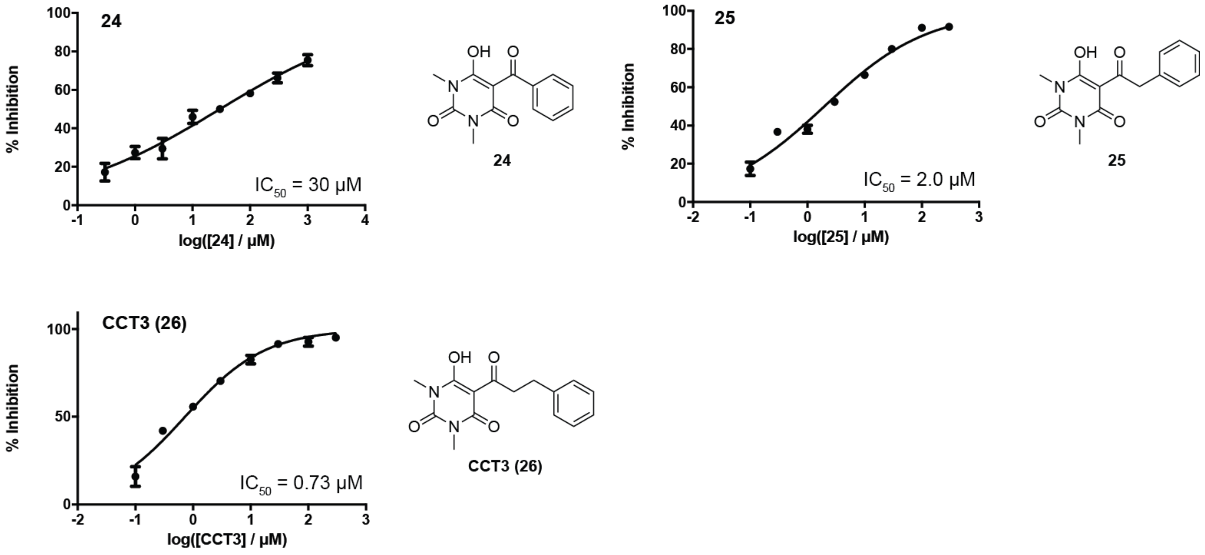

Figure S7. Determination of IC_{50} values for 24, 25, and CCT3 against OGFOD1. The inhibitory activity of serial dilutions of **24**, **25**, and **CCT3** (concentrations as indicated in figure) against OGFOD1 (1 µM) was tested under standard assay conditions (see Experimental section). The plotted data represent the mean percentage inhibition for each inhibitor concentration, performed in triplicate; the error bars indicate the standard deviation. The IC_{50} curve fitting was performed using GraphPad Prism 5. The standard error values associated with the logIC50 values calculated by Prism are 0.059 (**24**), 0.074 (**25**) and 0.059 (**CCT3**) (note that the data analysis was performed with µM inhibitor concentrations). Note that the inhibition observed for **24** and **25** did not plateau at the concentrations tested (possibly in part reflecting the peptide fragment of the natural protein substrates used in the assay), hence these IC_{50} values should be regarded as 'preliminary'.

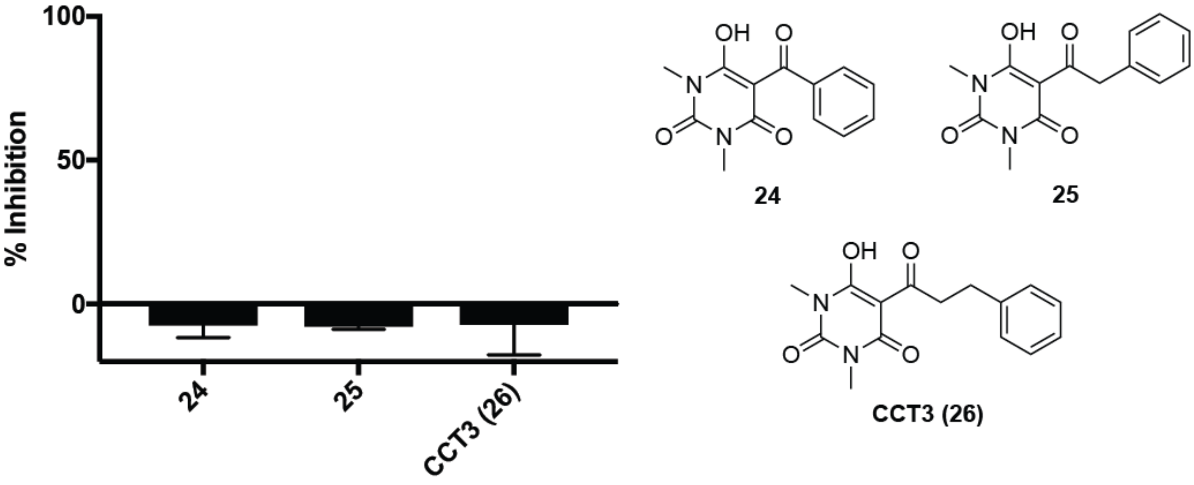

Figure S8. Lack of inhibition of PHD2 by selected triketone derivatives. The impact of **24**, **25**, and **CCT3** (**26**) (all 100 μM) on the hydroxylation activity of $PHD2_{181\text{-}426}$ (1 μM) was tested under standard assay conditions (see Experimental section). The plotted data represent the mean percentage inhibition for the experiment performed in triplicate; the error bars indicate the standard deviation.

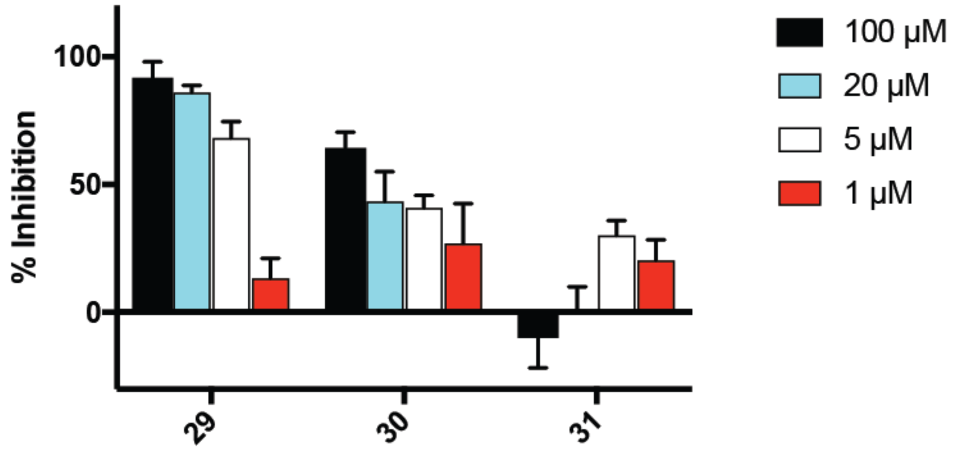

Figure S9. Impact of barbiturate *N*,*N′*-alkyl groups on OGFOD1 inhibition. The impact of *N*,*N′*-dimethyl (**29**), *N*,*N′*-diethyl (**30**), and *N*,*N′*-dicyclohexyl (**31**) barbiturate substitution on OGFOD1 inhibition. Inhibitors were tested at 100 µM (black), 20 µM (blue), 5 µM (white), and 1 µM (red) against OGFOD1 (1 µM) under standard assay conditions (see Experimental section). The plotted data represent the mean percentage inhibition for the experiment performed in triplicate; the error bars indicate the standard deviation.

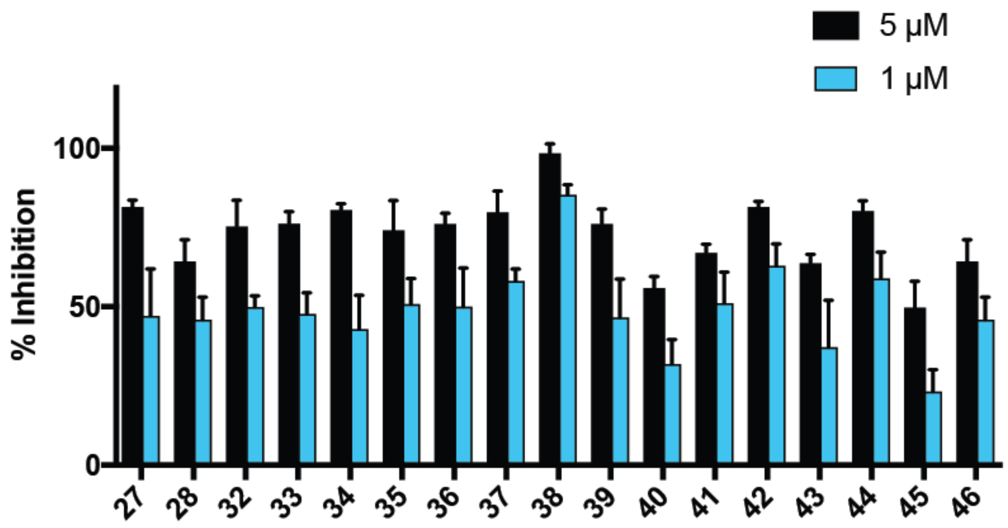

Figure S10. Inhibition of OGFOD1 by C-5-substituted barbiturate derivatives. The inhibitory activity of the synthesized panel of C-5 substituted *N*-methyl barbiturate derivatives was tested at 5 μM (black) and 1 μM (blue) against OGFOD1 (1 μM) under standard assay conditions (see Experimental section). The plotted data represent the mean percentage inhibition for the experiment performed in triplicate; the error bars indicate the standard deviation. The inhibitory potencies of the majority of these inhibitors resemble that observed for lead compound **CCT3** (**26**) during the determination of its IC_{50} value against OGFOD1 (Figure S7).

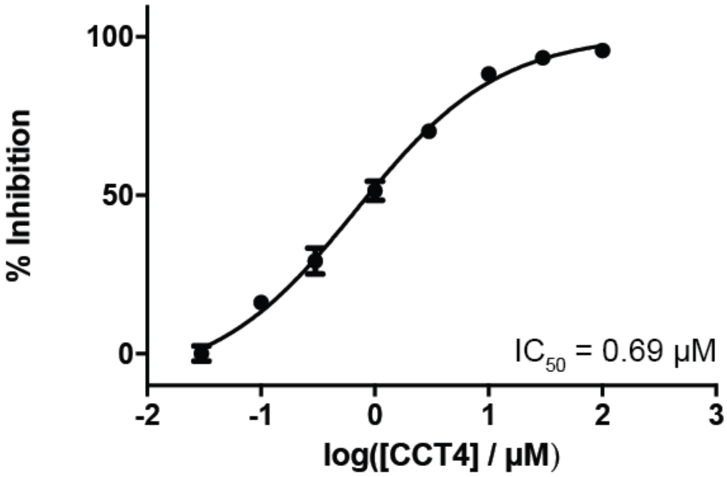

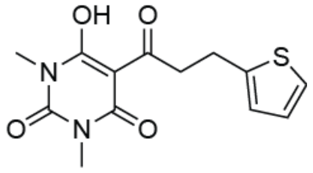

CCT4 (42)

Figure S11. Determination of IC_{50} values for CCT4 against OGFOD1. The inhibitory activity of serial dilutions of **CCT4** (concentrations as indicated in figure) against OGFOD1 (1 µM) was tested under standard assay conditions (see Experimental section). The plotted data represent the mean percentage inhibition for each inhibitor concentration, performed in triplicate, while the error bars indicate the standard deviation. The IC_{50} curve fitting was performed using GraphPad Prism 5. The standard error value associated with the logIC50 value calculated by Prism was 0.083 (note that the data analysis was performed with µM inhibitor concentrations).

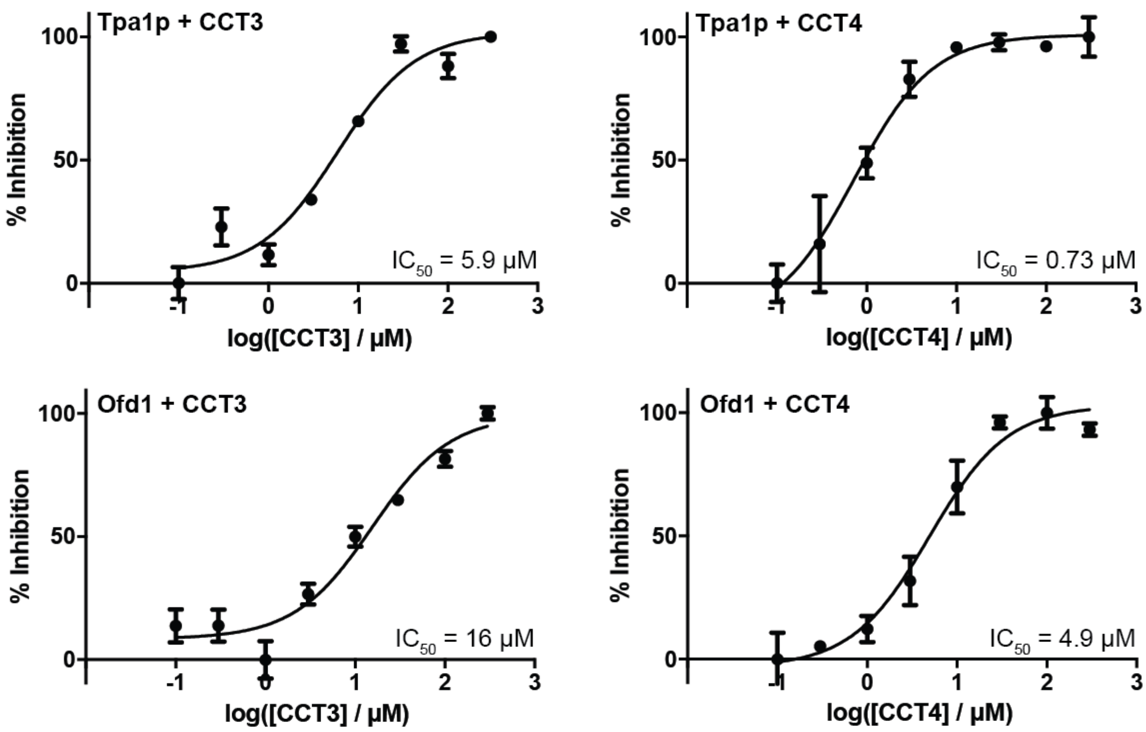

Figure S12. Determination of IC_{50} values for CCT3 and CCT4 against Tpa1p and Ofd1. The inhibitory activity of serial dilutions of **CCT3** and **CCT4** (concentrations as indicated in figure) against termination and polyadenylation protein 1 (Tpa1p; 2 µM) and Ofd1 (1 µM) was tested under standard assay conditions (see Experimental section). The plotted data represent the mean percentage inhibition for each inhibitor concentration, performed in triplicate, while the error bars indicate the standard deviation. The IC_{50} curve fitting was performed using GraphPad Prism 5. The standard error values associated with the logIC50 value calculated by Prism are 0.081 (**CCT3** and Tpa1p), 0.15 (**CCT4** and Tpa1p), 0.13 (**CCT3** and Ofd1), and 0.12 (**CCT4** and Ofd1) (note that the data analysis was performed with µM inhibitor concentrations).

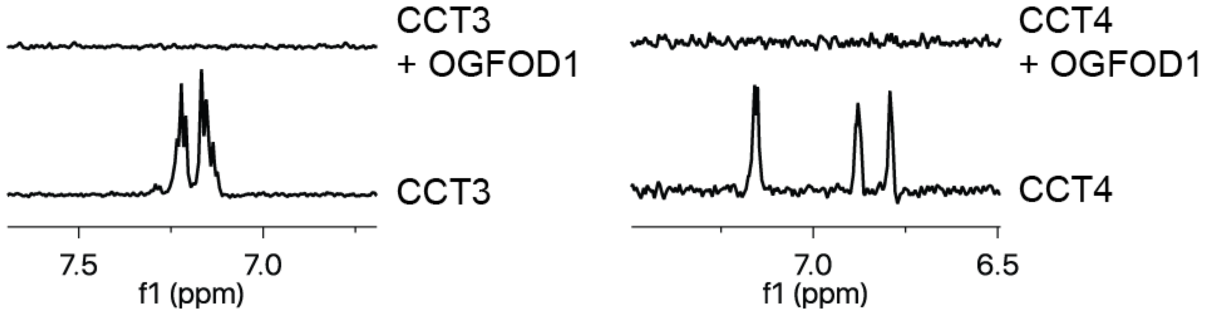

Figure S13. CPMG NMR analyses of CCT3 and CCT4 binding to OGFOD1. The binding of **CCT3** and **CCT4** to OGFOD1 was investigated using the Carr-Purcell-Meiboom-Gill (CPMG)-edited proton NMR spectra.[16] The disappearance of the aromatic signals (6-7 ppm) of **CCT3** or **CCT4** (30 μM) upon addition of OGFOD1 (30 μM) indicates a strong binding interaction. See the Experimental Section for further information on the conditions used.

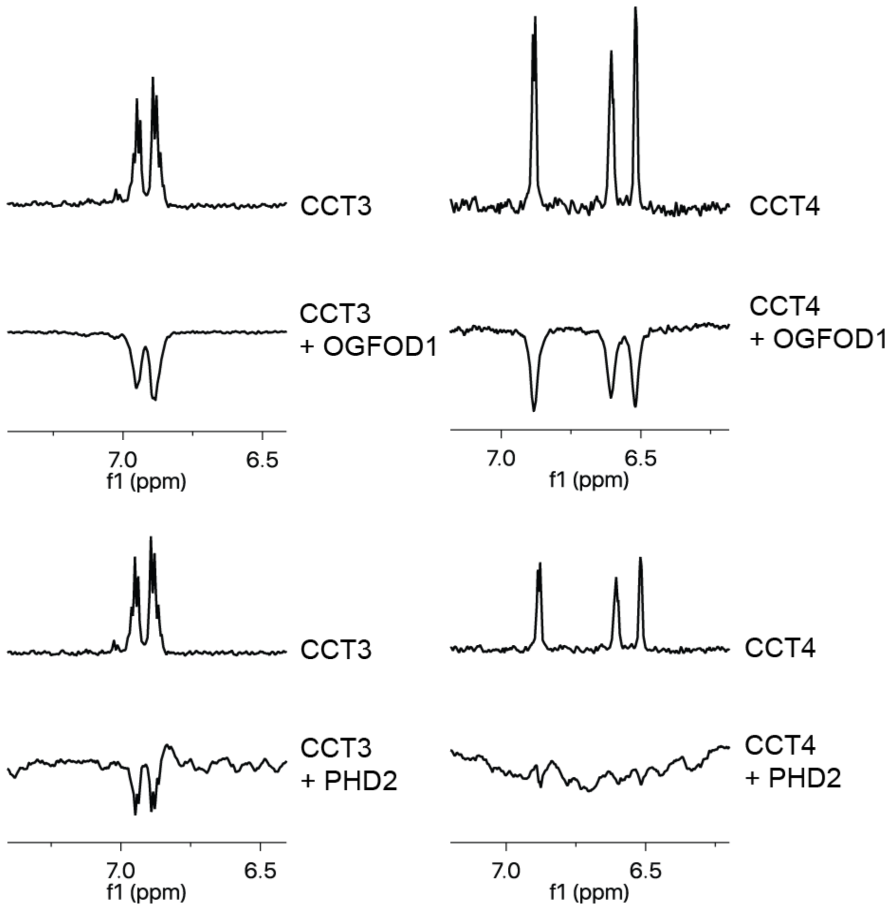

Figure S14. wLOGSY NMR analyses of CCT3 and CCT4 binding with OGFOD1 and PHD2. The binding of **CCT3** and **CCT4** to OGFOD1 and PHD2$_{181\text{-}426}$ was investigated using waterLOGSY NMR experiments. The strong inversion of the aromatic peaks (6-7 ppm) of **CCT3** or **CCT4** (120 µM) upon addition of OGFOD1 (15 µM) indicates a binding interaction. In these spectra, the positive signals represent free ligands in solution. The weakness or absence of such inverted peaks for **CCT3** and **CCT4** (400 µM) upon adding PHD2 (50 µM) indicates that the binding of these inhibitors to PHD2 is weaker when compared to OGFOD1. See the Experimental Section for further information on the conditions used.

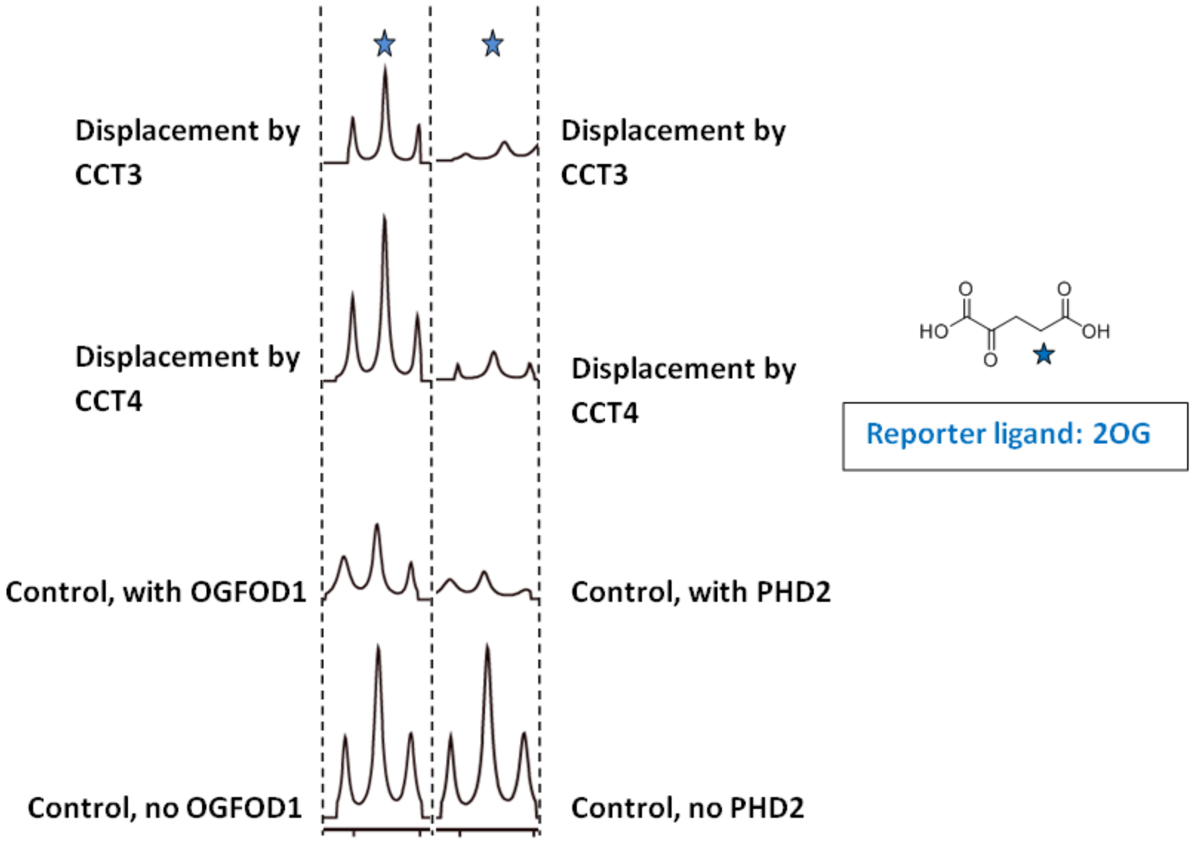

Figure S15. Single-concentration qualitative 2OG displacement as monitored by CPMG-edited ^{1}H NMR spectroscopy. When 2OG is bound to the protein, its corresponding ^{1}H NMR resonances are broadened and their intensities are decreased. In the presence of a competitive inhibitor, the reporter ligand is displaced from the protein binding site and its signal intensity is recovered. The spectra show the 2OG methylene resonance at 2.35 ppm. The assay mixture contained 10 μM of either *apo*-$PHD2_{181-402}$ or OGFOD1, 80 μM Zn(II), 10 μM 2OG, in 10 % D_2O and 90 % H_2O buffered with 50 mM Tris-D_{11}, pH 7.5 containing 0.02 % sodium azide. 2OG displacement was monitored following the addition of 70 μM inhibitor. See the Experimental Section for further information on the conditions used.

ADMET Assays

The solubility and metabolic stability of **CCT3** and **CCT4** was preliminarily evaluated using a S9 liver microsome assay (Cyprotex, UK). According to the assay, **CCT3** and **CCT4** had predicted intrinsic clearance values of 4.76 μL/min/mg protein and 7.72 μL/min/mg protein, respectively, and are thus classified as having low clearance (< 8.6 μL/min/mg protein).

The cell permeability of **CCT3** and **CCT4** were evaluated using an MDR1-MDCK *in vitro* permeability assay (Cyprotex, UK), the results of which suggest that both inhibitors are likely able to penetrate the blood-brain barrier. The permeability coefficients were determined to be 58.5 x 10^{-6} cm/s (**CCT3**), and 62.7 x 10^{-6} cm/s (**CCT4**), suggesting that brain uptake is likely.

References

[1] A. B. Pangborn, M. A. Giardello, R. H. Grubbs, R. K. Rosen, F. J. Timmers, *Organometallics* **1996**, *15*, 1518-1520.

[2] B. S. Jursic, D. M. Neumann, *Tetrahedron Lett.* **2001**, *42*, 8435-8439.

[3] S. Hu, S. Nian, K. Qin, T. Xiao, L. Li, X. Qi, F. Ye, G. Liang, G. Hu, J. He, Y. Yu, B. Song, *Chem. Pharm. Bull.* **2012**, *60*, 385-390.

[4] B. S. Jursic, D. M. Neumann, K. L. Bowdy, E. D. Stevens, *J. Heterocyclic Chem.* **2004**, *41*, 233-246.

[5] M. Temprado, M. V. Roux, F. Ros, R. Notario, M. Segura, J. S. Chickos, *J. Chem. Eng. D* **2010**, *56*, 263-268.

[6] M. Klikar, F. Bureš, O. Pytela, T. Mikysek, Z. Padělková, A. Barsella, K. Dorkenoo, S. Achelle, *New J. Chem.* **2013**, *37*, 4230-4240.

[7] S. Horita, J. S. Scotti, C. Thinnes, Y. S. Mottaghi-Taromsari, A. Thalhammer, W. Ge, W. Aik, C. Loenarz, C. J. Schofield, M. A. McDonough, *Structure* **2015**, *23*, 639-652.

[8] R. Chowdhury, K. K. Yeoh, Y. M. Tian, L. Hillringhaus, E. A. Bagg, N. R. Rose, I. K. Leung, X. S. Li, E. C. Woon, M. Yang, M. A. McDonough, O. N. King, I. J. Clifton, R. J. Klose, T. D. Claridge, P. J. Ratcliffe, C. J. Schofield, A. Kawamura, *EMBO Rep.* **2011**, *12*, 463-469.

[9] N. R. Rose, S. S. Ng, J. Mecinovic, B. M. R. Liénard, S. H. Bello, Z. Sun, M. A. McDonough, U. Oppermann, C. J. Schofield, *J. Med. Chem.* **2008**, *51*, 7053-7056.

[10] V. Bavetsias, R. M. Lanigan, G. F. Ruda, B. Atrash, M. G. McLaughlin, A. Tumber, N. Y. Mok, Y.-V. Le Bihan, S. Dempster, K. J. Boxall, *J Med Chem* **2016**, *59*, 1388-1409.

[11] J. A. Aguilar, M. Nilsson, G. Bodenhausen, G. A. Morris, *Chem. Commun.* **2012**, *48*, 811-813.

[12] I. K. Leung, M. Demetriades, A. P. Hardy, C. Lejeune, T. J. Smart, A. Szöllössi, A. Kawamura, C. J. Schofield, T. D. Claridge, *J. Med. Chem.* **2013**, *56*, 547-555.

[13] T.-L. Yeh, T. M. Leissing, M. I. Abboud, C. C. Thinnes, O. Atasoylu, J. P. Holt-Martyn, D. Zhang, A. Tumber, K. Lippl, C. T. Lohans, I. K. H. Leung, H. Morcrette, I. J. Clifton, T. D. W. Claridge, A. Kawamura, E. Flashman, X. Lu, P. J. Ratcliffe, R. Chowdhury, C. W. Pugh, C. J. Schofield, *Chem. Sci.* **2017**, *8*, 7651-7668.

[14] H. J. Choi, B. J. Song, Y. D. Gong, W. J. Gwak, Y. Soh, *Br J Pharmacol* **2008**, *154*, 114-125.

[15] R. Chowdhury, M. A. McDonough, J. Mecinović, C. Loenarz, E. Flashman, K. S. Hewitson, C. Domene, C. J. Schofield, *Structure* **2009**, *17*, 981-989.

[16] P. J. Hajduk, E. T. Olejniczak, S. W. Fesik, *J. Am. Chem. Soc.* **1997**, *119*, 12257-12261.
